# Structure and ecological function of the soil microbiome associated with ‘Sanghuang’ mushrooms suffering from fungal diseases

**DOI:** 10.1186/s12866-023-02965-z

**Published:** 2023-08-12

**Authors:** Weifang Xu, Tao Sun, Jiahui Du, Shuqing Jin, Ying Zhang, Guofa Bai, Wanyu Li, Dengke Yin

**Affiliations:** 1 Anhui Province Key Laboratory of Research & Development of Chinese Medicine, School of Pharmacy, Anhui University of Chinese Medicine, Hefei, 230012 China; 2 School of Life Science and Technology, China Pharmaceutical University, Nanjing, 211198 China

**Keywords:** ‘Sanghuang’ mushroom, Fungal diseases, Soil microbiome, Biocontrol agents

## Abstract

**Background:**

The most serious challenges in medicinal ‘Sanghuang’ mushroom production are the fungal diseases caused by various molds. Application of biological agents has been regarded as a potential crop disease management strategy. Here, the soil microbiome associated with ‘Sanghuang’ mushroom affected by fungal diseases grown under field cultivation (FC) and hanging cultivation (HC) was characterized using culture-dependent and culture-independent methods.

**Results:**

A total of 12,525 operational taxonomic units (OTUs) and 168 pure cultures were obtained using high-throughput sequencing and a culture-dependent method, respectively. From high-throughput sequencing, we found that HC samples had more OTUs, higher α-diversity, and greater microbial community complexity than FC samples. Analysis of β-diversity divided the soil microbes into two groups according to cultivation mode. *Basidiomycota* (48.6%) and *Ascomycota* (46.5%) were the two dominant fungal phyla in FC samples, with the representative genera *Trichoderma* (56.3%), *Coprinellus* (29.4%) and *Discosia* (4.8%), while only the phylum *Ascomycota* (84.5%) was predominant in HC samples, with the representative genera *Discosia* (34.0%), *Trichoderma* (30.2%), *Penicillium* (14.9%), and *Aspergillus* (7.8%). Notably, *Trichoderma* was predominant in both the culture-independent and culture-dependent analyses, with *Trichoderma* sp. FZ0005 showing high host pathogenicity. Among the 87 culturable bacteria, 15 exhibited varying extents of antifungal activity against *Trichoderma* sp. FZ0005, with three strains of *Bacillus* spp. (HX0037, HX0016, and HX0039) showing outstanding antifungal capacity.

**Conclusions:**

Overall, our results suggest that *Trichoderma* is the major causal agent of ‘Sanghuang’ fungal diseases and that *Bacillus* strains may be used as biocontrol agents in ‘Sanghuang’ cultivation.

**Supplementary Information:**

The online version contains supplementary material available at 10.1186/s12866-023-02965-z.

## Introduction

A precious basidiomycete fungus, *Inonotus baumii* (formerly *Phellinus baumii*), has been widely utilized in China, Japan and other Asian countries as a traditional Chinese medicinal mushroom [1, 2]. In China, *I. baumii* is commonly called ‘Sanghuang’ mushroom, a yellow fungus that grows on mulberry. It is regarded as being advantageous to human health due to its high biological activities, including antitumor, anti-inflammation, and antioxidation effects [3–5]. Numerous studies have documented that natural compounds such as polysaccharides, flavones, and ergosterol are the bioactive metabolites responsible for the medicinal and gastronomic value of ‘Sanghuang’ [6, 7]. In recent years, indoor artificial cultivation of ‘Sanghuang’ mushroom has been developed and practiced on a large scale in China [8]. However, various fungal diseases caused by molds such as *Trichoderma*, *Penicillium*, and *Mucor* [9, 10] significantly impair the quality and yield of ‘Sanghuang’ mushroom, resulting in economic losses.

**Fig. 1:**
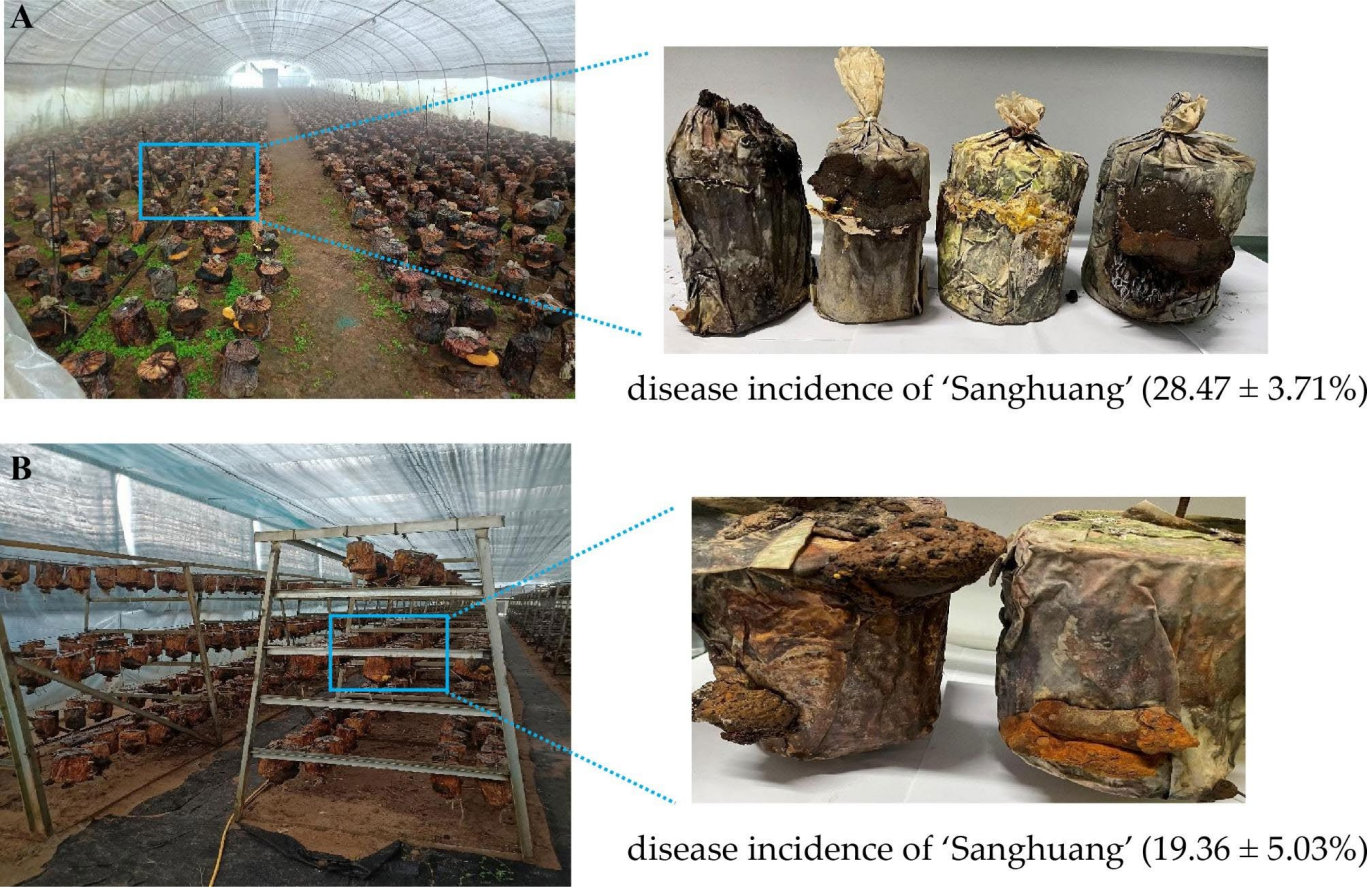
Growth status of ‘Sanghuang’ mushrooms grown under different cultivation modes **(A)** the field cultivation (FC) mode; **(B)** the hanging cultivation (HC) mode. Disease incidence was calculated as a percentage of five replicates. The results are presented as the mean plus standard deviation

Numerous farmlands in China have been outfitted with greenhouses for the artificial cultivation of ‘Sanghuang’ mushroom [11, 12]. Plantations in Jinzhai County, Anhui Province, can produce approximately 20 tons of ‘Sanghuang’ mushroom annually. Generally, field cultivation (FC) and hanging cultivation (HC) are the two common modes of wood-inhabiting ‘Sanghuang’ mushroom cultivation. Analogous to other large-scale mushroom production [13, 14], ‘Sanghuang’ mushroom cultivation has extremely strict environmental requirements, including temperature and humidity control and appropriate lighting and ventilation. Unfortunately, these environmental conditions in the greenhouse are also conducive to the spore germination and mycelial growth of many molds. When investigating the growth status of ‘Sanghuang’ mushroom in Jinzhai County Anhui Province, we discovered that ‘Sanghuang’ mushroom grown under FC and HC experienced varying extents of fungal diseases (disease incidence: 28.47% for FC, 19.36% for HC) under control conditions (Fig. 1). These findings inspired us to investigate the major causal agents of ‘Sanghuang’ fungal diseases and expand our search for an ecofriendly prevention and control strategy.

Soil is a complex environment that can contain many microbial taxa, which are key components of both natural and managed ecosystems [15]. Soil microbes play important roles in numerous ecological processes, such as cycling of materials and energy, soil structure stability, and fertility [16]. Numerous studies have also revealed that there are close symbiotic relationships between soil microbes and mushrooms. For example, many soil microbes can attack fruiting bodies at various stages of mushroom growth, producing distinct disease symptoms [17, 18], while certain beneficial species such as *Pseudomonas* and *Bacillus* can significantly improve crop total fresh matter yield and shorten harvesting time [19, 20]. In recent decades, studies on soil microbial community structure have been reported for many crops [21, 22], while ‘Sanghuang’ mushroom has mostly been studied for its pharmacodynamic elements and their effects [23–25]. However, information on the soil microbiome associated with ‘Sanghuang’ mushroom remains scarce.

Environmental microorganisms have typically been isolated and analyzed using conventional culture-dependent methods [26, 27]. High-throughput sequencing technology has recently provided a new approach, culture-independent protocols with the benefits of no cultivation and high resolution, to investigate the diversity of the environmental microbial community [28–31]. A more thorough evaluation of the composition of the microbial community can be performed by using both culture-dependent and culture-independent methods [32, 33]. Therefore, the aims of the present study were to (i) characterize the soil microbiome associated with ‘Sanghuang’ mushroom by using both culture-dependent and culture-independent methods and (ii) screen for potential biocontrol bacteria with antifungal activity against the major pathogens causing fungal diseases in ‘Sanghuang’ mushroom.

## Materials and methods

### Sampling site and sample collection

The sampling site of this study was located at the artificial Chinese medicine cultivation base in Jinzhai County, Anhui Province, China (31°46’28”N, 115°54’58”E), which is one of the major ‘Sanghuang’-producing regions in China. In September 2021, two types of soil samples were collected from FC and HC systems, which were the growth habitats of 2-year-old ‘Sanghuang’ mushroom (Fig. 1). In addition, the disease incidence of ‘Sanghuang’ was investigated at the time of sampling. For each cultivation mode, the total number of ‘Sanghuang’ mushrooms and the number of mushrooms suffering from fungal diseases in five randomized plots were recorded. The disease incidence of each cultivation mode was calculated as follows: disease incidence (%) = the number of ‘Sanghuang’ mushrooms suffering from fungal diseases/the total number of mushrooms examined×100. For each soil type in this study, five sampling sites were selected for each mushroom cultivation mode, and using tweezers, we collected approximately 10 g of soil each from the surface and at a depth of 5 cm. All samples were then placed into sterile polythene bags, immediately transported back to the laboratory on ice and stored at -80 °C until further processing.

### Sample preparation and physicochemical analysis

The soil samples collected as described above were sieved through a 100-mm sieve to remove stones, grass, and other materials. Following sieving, the soil was subjected to physicochemical analysis. The electrical conductivity (EC), potential of hydrogen (pH), available nitrogen (AN), available phosphorus (AP), and available potassium (AK) were measured using a PR-3001-TRREC-N01 analyzer (ENVIRONMENTAL MONITORING Co., China) following the manufacturer’s instructions.

### Analysis of the FC and HC microbiomes

To ensure the accuracy of soil microbiome analysis, both culture-independent and culture-dependent methods were used in this study. The culture-independent method was based on ITS and 16 S rDNA amplicon sequencing performed on the NovaSeq platform. Both types of soil samples (FC and HC) were sequenced with 5 replicates each. Culture-dependent methods based on plating isolation and microbial identification techniques were also used to analyze the microbial communities under FC and HC.

### Culture-independent molecular analysis

Total genomic DNA was extracted from soil samples using a modified cetyltrimethylammonium bromide-based (CTAB) protocol [34]. The final DNA concentration and quality were assessed using a NanoDrop 2000 UV-Spectrophotometer (Thermo Scientific, Wilmington, USA). The ITS1 region was amplified using the primer set ITS1F (5’-CTTGGTCATTTAGAGGAAGTAA-3’) and ITS1R (5’-GCTGCGTTCTTCATCGATGC-3’), while the V3-V4 variable region of the 16S rRNA gene was amplified using the primer set 341F (5’-CCTAYGGGRBGCASCAG-3’) and 806R (5’-GGACTACNNGGGTATCTAAT-3’). The fungal PCRs were performed in a final volume of 30 µL containing 15 µL of 2× Phusion Master Mix, 1.5 µL of each primer (2 µM), 10 µL of gDNA (1 ng/µL), and 2 µL of H_2_O. The bacterial PCRs were performed in a final volume of 30 µL containing 15 µL of 2× Phusion Master Mix, 1 µL of each primer (0.2 µM), 10 µL of gDNA (1 ng/µL), and 3 µL of H_2_O. Both fungal and bacterial PCRs were conducted using the following conditions: 98 °C for 1 min; 30 cycles of 98 °C for 10 s, 50 °C for 30 s and 72 °C for 30 s; and a final extension at 72 °C for 5 min. The PCR product was detected and qualified by 2% agarose gel electrophoresis. PCR products were mixed in equidensity ratios. Then, mixed PCR products were purified with a Qiagen Gel Extraction Kit (Qiagen, Germany) according to the manufacturer’s instructions. The library was established using the TruSeq® DNA PCR-Free Sample Preparation Kit (Illumina, San Diego, CA, USA), followed by Qubit quantification and library detection. If qualified, sequencing was performed using NovaSeq 6000 PE250, and bioinformatics analysis was performed using the platform of Wekemo Tech Group Co., Ltd. (Shenzhen, China) (https://www.bioincloud.tech/). Raw sequencing data obtained from this study were deposited in the NCBI Sequence Read Archive (SRA) database.

### Culture-dependent microbiological analysis

The method used to isolate soil microbes was based on procedures described by Cai et al. [35]. Briefly, 1 g of soil was added to 100 mL of sterile water in a 250 mL flask and shaken on a shaking table for 10 min to create a suspension that was serially diluted 10-fold. Subsequently, 100 µL suspensions of 1:1,000 to 1:100,000 dilutions were spread on water agar (WA), Gause’s agar (GA), and potato dextrose agar (PDA) media plates and incubated at 30 °C. The cultures were observed daily, and colonies with different morphological characteristics were purified as they appeared on the plates. The purified isolates of fungi and bacteria were stored in 20% and 30% glycerol solutions at -80 °C, respectively.

Taxonomic identification of the obtained isolates was performed as previously described by Xu et al. [36, 37]. Classification of the fungi and bacteria was based on ITS and 16 S rRNA gene sequencing, respectively. Fungal DNA samples were amplified using the primers ITS1 (5’-TCCGTAGGTGAACCTGCGG-3’)/ITS4 (5’-TCCTCCGCTTATTGATATGC-3’), and bacterial DNA samples were amplified using the primers 27 F (5’-AGAGTTTGATCCTGGCTCAG-3’)/1492R (5’-GGTTACCTTGTTACGACTT-3’) [38, 39]. The PCRs were performed in a final volume of 25 µL consisting of 12.5 µL of Taq PCR Master Mix, 1.0 µL of Primer-F, 1.0 µL of Primer-R, 1.0 µL of DNA template and 9.5 µL of ddH_2_O [40]. The fungal PCRs were conducted using the following conditions: 95 °C for 5 min; 30 cycles of 94 °C for 50 s, 52 °C for 1 min and 72 °C for 55 s; and a final extension at 72 °C for 10 min. The bacterial PCRs were conducted using the following conditions: 95 °C for 7 min; 30 cycles of 94 °C for 30 s, 50 °C for 45 s and 72 °C for 1 min; and a final extension at 72 °C for 8 min. Both PCR-amplified products were purified with a DNA Clean & Concentrator™-5 Kit (Zymo Research, USA) and then Sanger sequenced at Sangon Biotechnology Co., Ltd., Shanghai, China. The generated gene sequences were uploaded to the NCBI database (National Center for Biotechnology Information, https://blast.ncbi.nlm.nih.gov/Blast.cgi), and then, the Basic Local Alignment Search Tool (BLAST) was used to determine sequence homology with closely related taxa. All of the microbial strains were classified using the taxonomic database of the NCBI. All ITS/16S rRNA gene sequence information obtained in this study was submitted to the NCBI by the Bankit tool.

### Pathogenicity test of the pathogen and screening of antagonistic bacteria

The pathogenicity of *Trichoderma* sp. FZ0005, which was isolated from the soil of ‘Sanghuang’ mushroom, was verified using Koch’s postulates (Supplementary Fig. S1), and the FZ0005 strain was used as the target to assay the biocontrol potential of all soil bacteria. The antifungal activity of all the bacterial isolates was qualitatively assayed by the well diffusion technique [36]. Briefly, agar discs (5 mm) of the pathogenic fungi were placed at the centers of fresh potato dextrose agar (PDA) plates, and wells were punched 30 mm from the centers of the agar discs. The fermentation supernatant of the bacterial isolate was then added to the wells. After 4 days of incubation at 26 °C, the diameters of the fungal inhibition zones (Di) were measured to assess the antifungal activity of the test bacteria. All these treatments were performed in triplicate.

### Statistical analyses

All soil physicochemical analysis data were analyzed using SPSS software (Version 26.0), and the results are presented as the mean ± standard deviation. Then, a t test was used to detect differences between two groups. For culture-independent molecular analysis, the QIIME tools import program was used to import FASTQ files of raw data in the format operated by QIIME2. The QIIME2 dada2 plugin was used for quality filtering and trimming, denoising, merging, and identifying and removing chimeras, and then the feature table of amplicon sequence variants (ASVs) was obtained [41]. ASV sequences were aligned to a pretrained UNITE 8.2 99% database (the database is trimmed to the ITS1 region bound by the ITS1F/ITS1R primer pair) for fungi, and the GREENGENES 13_8 99% database (the database is trimmed to the V3-V4 region bound by the 338 F/806R primer pair) for bacteria to generate the taxonomy table using the QIIME2 feature-classifier plugin. The QIIME2 feature-table plugin was used to filter any contaminating mitochondrial and chloroplast sequences. Rarefaction curves were generated to confirm the reliability of the sequencing data from samples at the 99% identity level. A Venn diagram was used to show the common and unique microbial OTUs among samples. Alpha-diversity indices, including the Chao and Shannon indices, were calculated by the QIIME2 core-diversity plugin, and a t test was used to assess the differences in alpha-diversity indices between groups. The β-diversity of microbial communities across samples was visualized using principal coordinate analysis (PCoA) based on Bray‒Curtis distances [42], and we detected significant differences in community structure by permutational multivariate analysis of variance (PERMANOVA). Moreover, a clustering heatmap was used to show the similarity of microbial communities between groups. Network analysis was used to demonstrate interspecies relationships in order to evaluate the complexity of the interactions among the microbial taxa [43]. The potential KEGG Ortholog (KO) functional profiles of microbial communities were predicted with PICRUSt [44], and then ANOVA and Duncan analysis were used to assess functional differences between groups. A heatmap was used to investigate the relationships between soil physicochemical properties and total soil microbial communities under the two cultivation modes. Additionally, the composition of soil microbial communities (the top 20 species in terms of relative abundance) was inferred based on ITS/16S rDNA amplicon sequencing, and Faith’s phylogenetic diversity index was used to calculate the distance from the OTU of each soil sample to the phylogenetic tree. The LEfSe method was used to detect differences in abundance of the soil microbiome between groups.

## Results

### Characterization of soil physicochemical properties

The soil physicochemical properties under FC and HC were compared, as shown in Table 1. The pH values of the two types of soil were roughly equivalent (6.1 for FC, 6.2 for HC), and other indicators (e.g., EC, AN, AP, and AK) of the FC soil samples were slightly higher than those of the HC soil samples. However, there were no significant differences in any of the measured indices between the FC and HC groups (Table 1).

**Table 1:** Soil physicochemical properties of ‘Sanghuang’ mushrooms

Sample	EC (us/cm)	pH	AN (mg/kg)	AP (mg/kg)	AK (mg/kg)
FC	206.4 ± 3.6	6.1 ± 0.3	17.6 ± 0.5	24.0 ± 0.7	60.2 ± 1.3
HC	203.0 ± 6.3	6.2 ± 0.1	17.4 ± 0.5	23.4 ± 0.5	59.0 ± 1.6
*p*	0.3	0.2	0.6	0.2	0.2

Note: FC and HC represent soil samples from the field cultivation mode and hanging cultivation mode, respectively; EC, pH, AN, AP and AK represent electrical conductivity, potential of hydrogen, available nitrogen, available phosphorus, and available potassium, respectively

### High-throughput amplicon sequencing

**Fig. 2:**
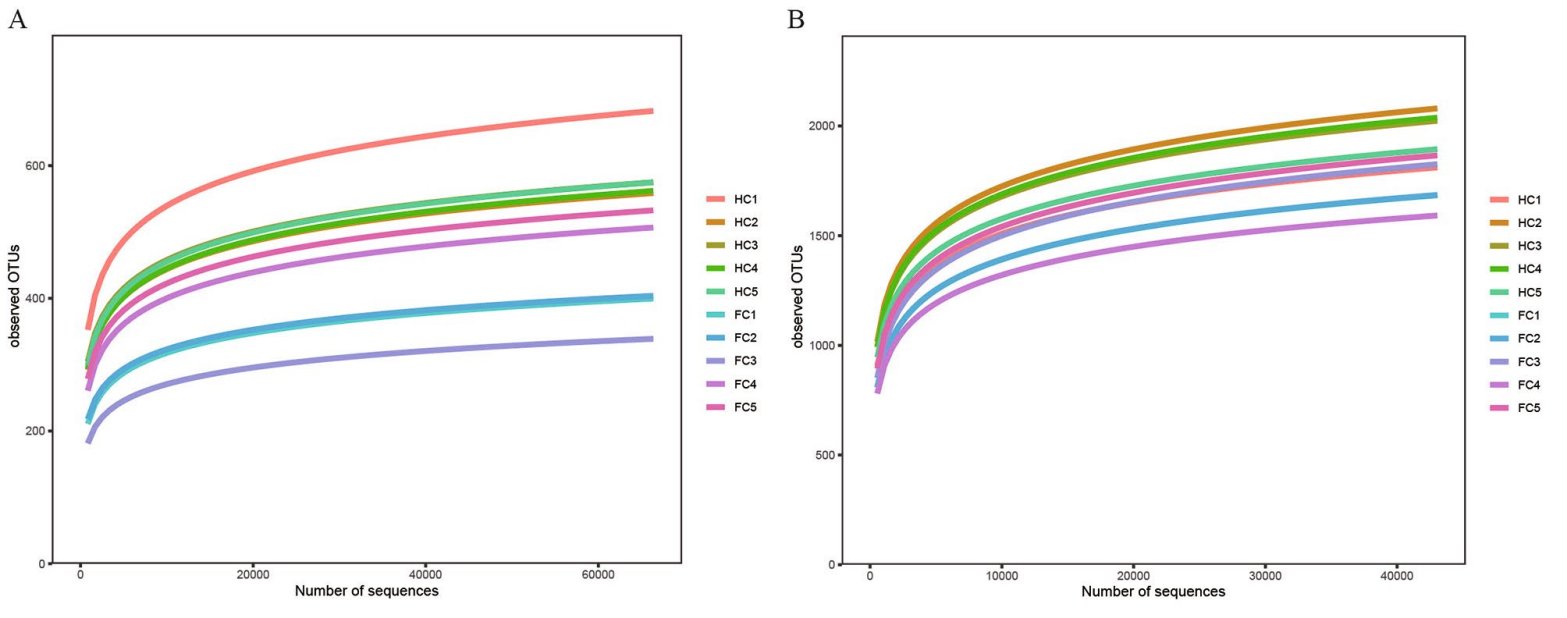
Rarefaction curves of observed OTUs at 99% sequence similarity for all soil samples. **(A)** fungi; **(B)** bacteria. FC and HC represent soil samples from the field cultivation mode and hanging cultivation mode, respectively; numbers 1 to 5 refer to the replicates of each sample

**Fig. 3:**
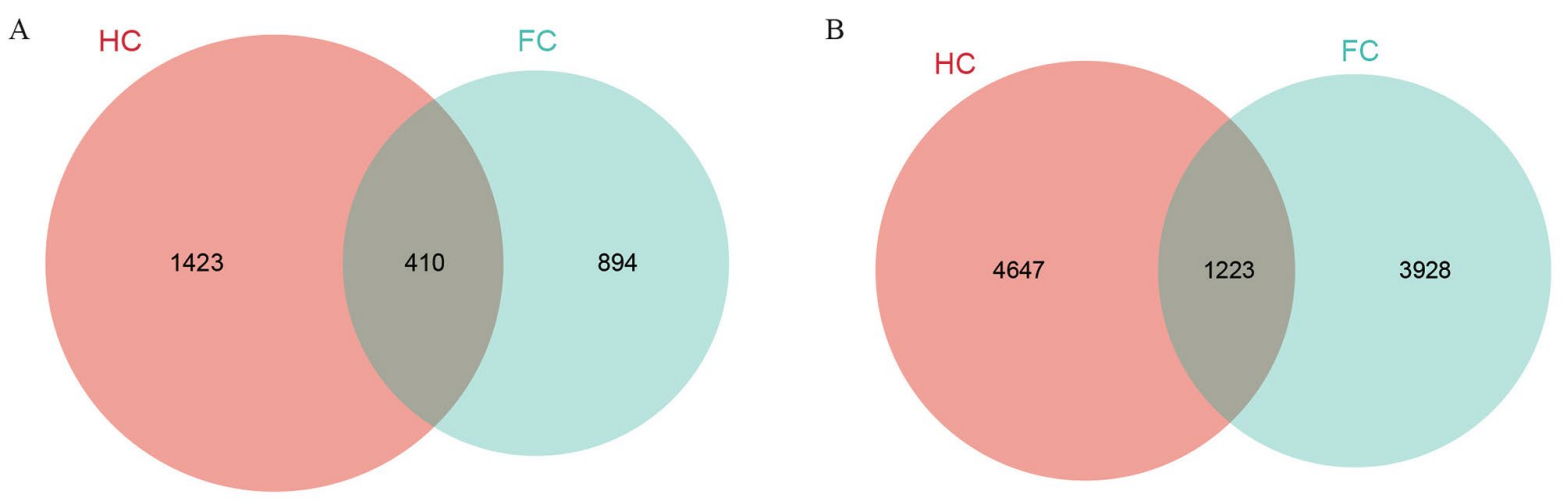
Microbial OTU Venn diagram of different soil samples. **(A)** fungi; **(B)** bacteria. FC and HC represent soil samples from the field cultivation mode and hanging cultivation mode, respectively; the number of OTUs shown in the Venn diagram is the total number of OTU species for the 5 soil replicates

After quality control and filtering, a total of 711,190 ITS and 534,466 16 S rDNA effective sequences were obtained from 10 DNA samples, including two types of soil samples and five replicates (Supplementary Table S1). All rarefaction curves tended to reach a plateau, suggesting that a reasonable sequencing depth was attained (Fig. 2) [45]. The common and unique microbial OTUs in different samples are shown in the Venn diagram (Fig. 3). The number of OTUs differed between the FC and HC samples, where the total number of OTUs in HC samples was obviously higher than that in FC samples, regardless of fungi (1833 for HC, 1304 for FC) or bacteria (5870 for HC, 5151 for FC). A similar trend also appeared in the number of unique OTUs between the two samples.

### Diversity estimation

**Fig. 4:**
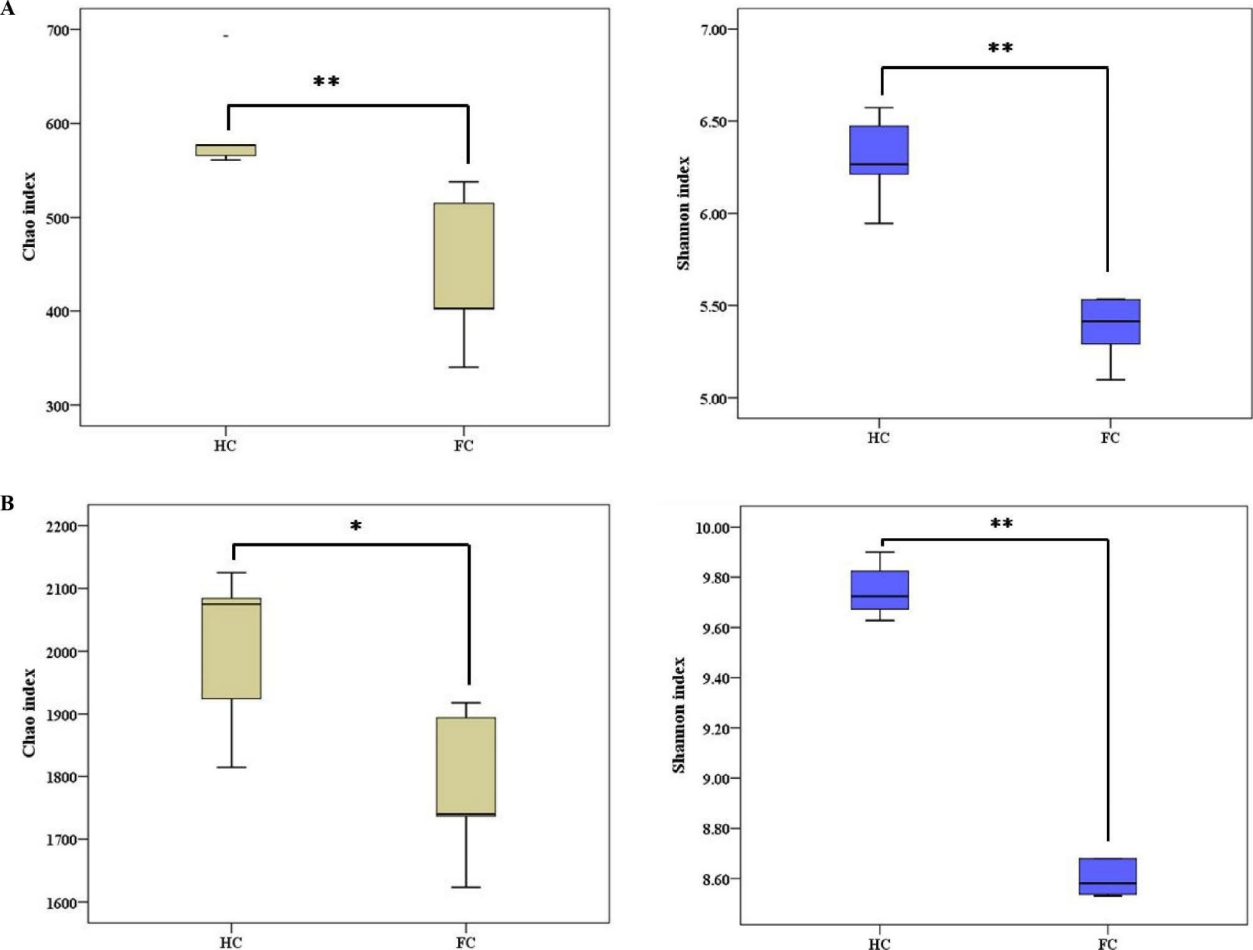
α-Diversity analysis of the soil microbial community of ‘Sanghuang’ mushroom. **(A)** Chao and Shannon indices for fungi; **(B)** Chao and Shannon indices for bacteria. FC and HC represent soil samples from the field cultivation mode and hanging cultivation mode, respectively; * represents ‘*p* < 0.05’, ** represents ‘*p* < 0.01’

The Chao index and Shannon index calculated for microbial OTUs were used to further compare the richness and diversity of the soil microbial communities between the FC and HC samples, respectively [46]. For fungi, the average value of both the Chao index and the Shannon index in the HC samples exhibited a clear upward trend compared to that in the FC samples (Chao: HC (594.80) > FC (439.60), Shannon: HC (6.29) > FC (5.45) (*p* < 0.01)) (Fig. 4A and Supplementary Table S2). For bacteria, the average value of both the Chao index and the Shannon index in HC samples was also significantly higher than that in FC samples (Chao: HC (2004.62) > FC (1782.47) (*p* < 0.05), Shannon: HC (9.75) > FC (8.67) (*p* < 0.01)) (Fig. 4B and Supplementary Table S2). This phenomenon indicated that HC samples harbored a higher α-diversity (within-sample diversity) than FC samples.

**Fig. 5:**
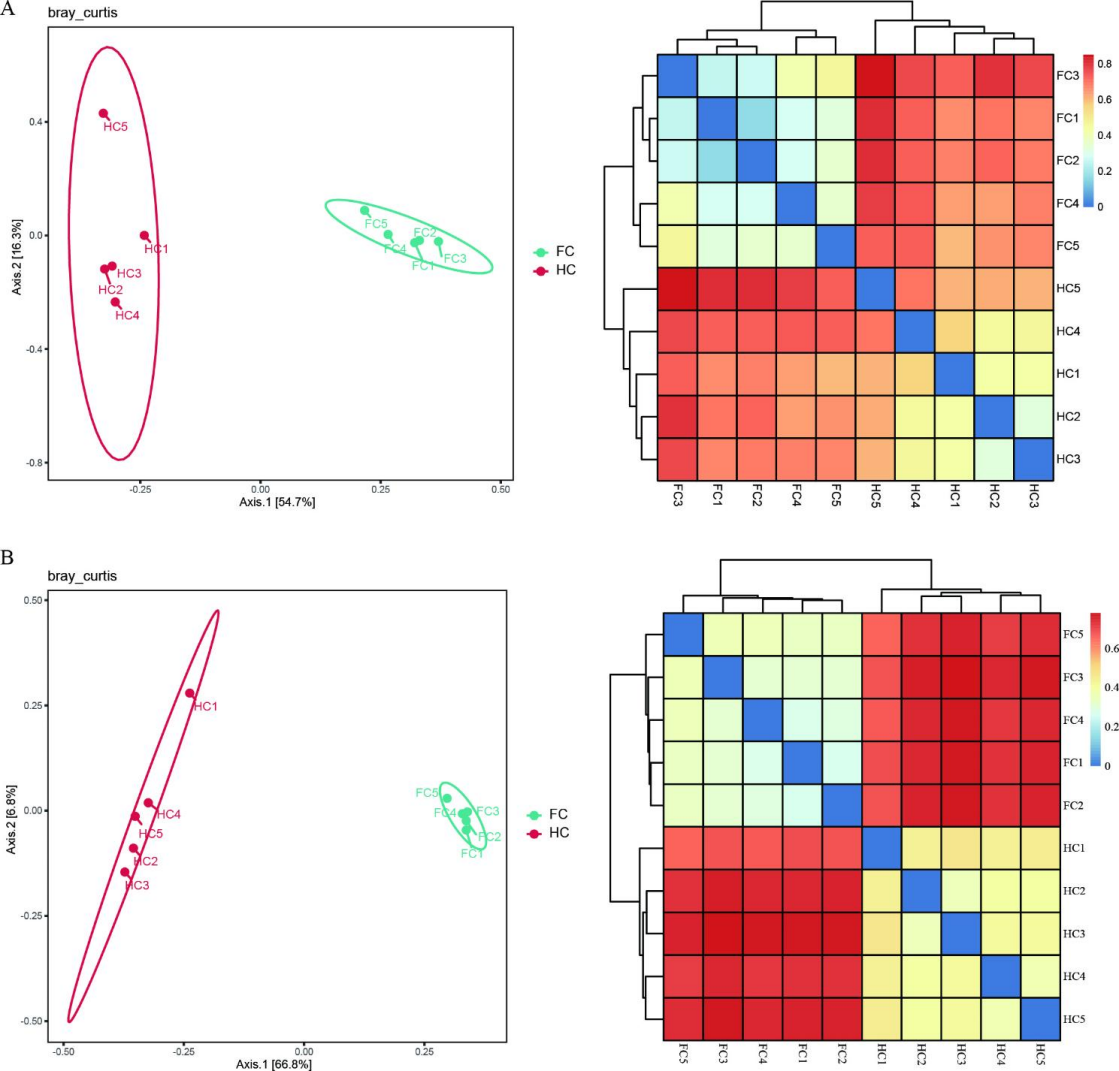
PCoA and clustering heatmap analysis of different soil samples for the fungal community **(A)** and bacterial community **(B)**. FC and HC represent soil samples from the field cultivation mode and hanging cultivation mode, respectively; numbers 1 to 5 refer to the replicates of each sample

To reveal differences among samples, β-diversity analysis was assessed using Bray‒Curtis distances at the OTU level, including unconstrained PCoA, which was used to reflect the difference in community structure between groups based on the spatial distance among samples [47], and the clustering heatmap. Both PCoA and clustering heatmap analyses indicated that samples collected under FC and HC differed in their microbial communities, irrespective of soil fungi or soil bacteria (Fig. 5). In addition, a significant difference in community structure was revealed by PERMANOVA (fungi: *p* = 0.007, bacteria: *p* = 0.008) (Supplementary Table S3).

### Network structure of the soil microbiome

**Fig. 6:**
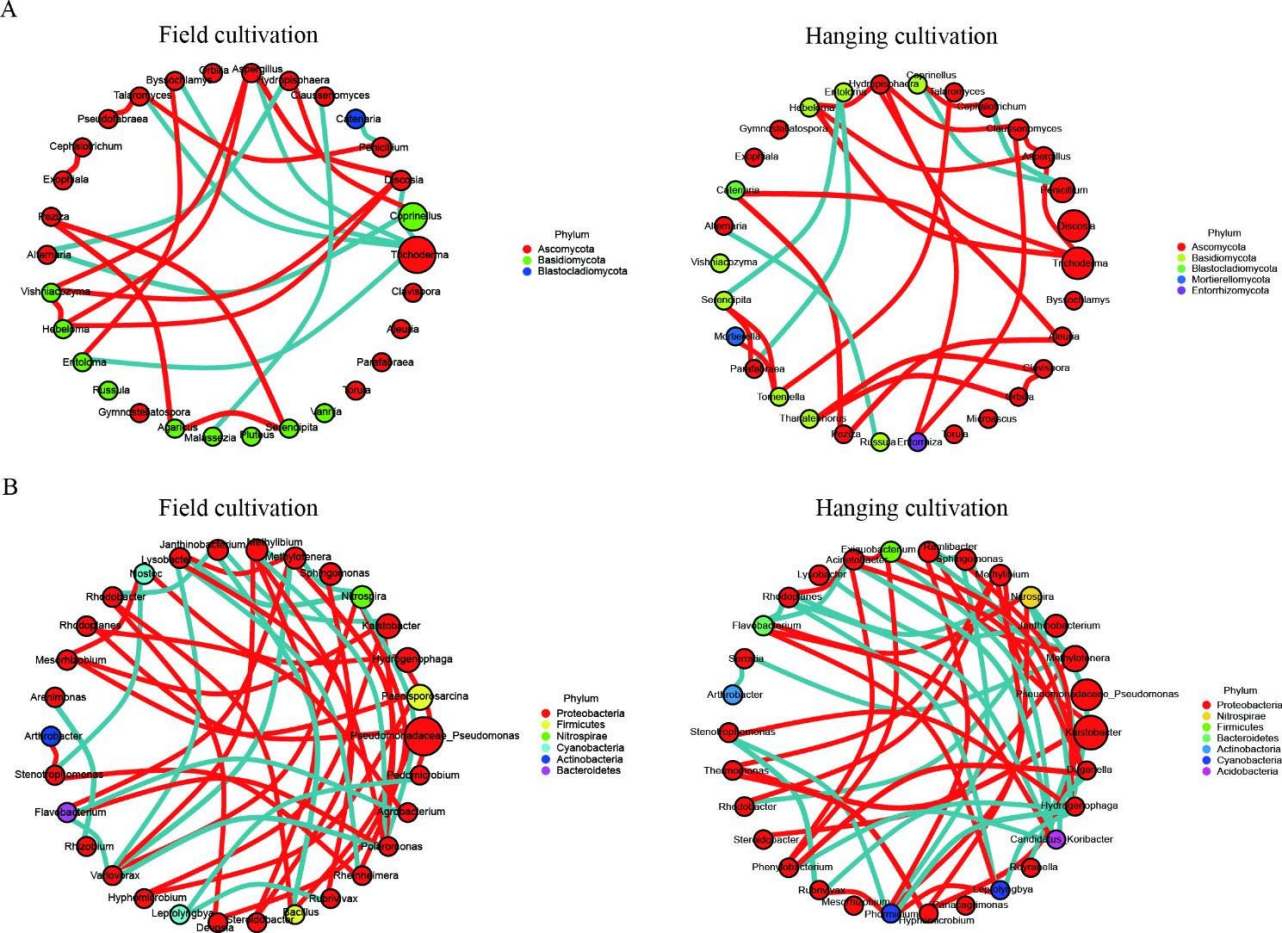
Species correlation networks of soil microorganisms at the genus level for fungal **(A)** and bacterial communities **(B)**. The size of the nodes in the figure represents the species abundance, and their color represents different phyla; the red and green connections represent significant positive and significant negative correlations, respectively; the more lines, the more closely the species are connected

Species correlation network analysis of soil microorganisms was conducted to demonstrate the species interaction of soil microorganisms in the two cultivation modes. The species correlation networks of soil microorganisms in HC samples at the genus level were more complex than those in FC samples (number of edges: for fungi, HC (25) > FC (23); for bacteria, HC (49) > FC (44)), and the species in HC were more closely related (Fig. 6 and Supplementary Table S4). The dominant fungal genus in terms of relative abundance in both FC and HC was *Trichoderma*, which was mainly involved in the establishment of soil fungal correlation networks in the ‘Sanghuang’ cultivation environment. In the FC samples, *Trichoderma* was significantly negatively correlated with the other five genera (*Discosia*, *Aspergillus*, *Byssochlamys*, *Talaromyces*, and *Entoloma*) (*p* < 0.05). *Aspergillus*, *Hydropisphaera*, and *Hebeloma* had significant positive correlations with *Trichoderma* in HC samples (*p* < 0.05). For the bacterial community correlations, *Methylotenera*, *Variovorax*, and *Polaromonas* were mainly involved in the establishment of bacterial correlation networks in FC samples. *Methylotenera* had significant negative correlations with *Nitrospira*, *Methylibium*, and *Variovorax* and significant positive correlations with *Lysobacter*, *Flavobacterium*, and *Polaromonas* (*p* < 0.05). *Phormidium* and *Hydrogenophaga* were mainly involved in the establishment of bacterial correlation networks in HC samples. *Hydrogenophaga* had significant negative correlations with *Nitrospira*, *Ramlibacter*, *Rubrivivax*, *Phormidium*, and *Leptolyngbya* and was significantly positively correlated with *Sphingomonas* and *Stenotrophomonas* (*p* < 0.05).

### Functional prediction of the soil microbiome

**Fig. 7:**
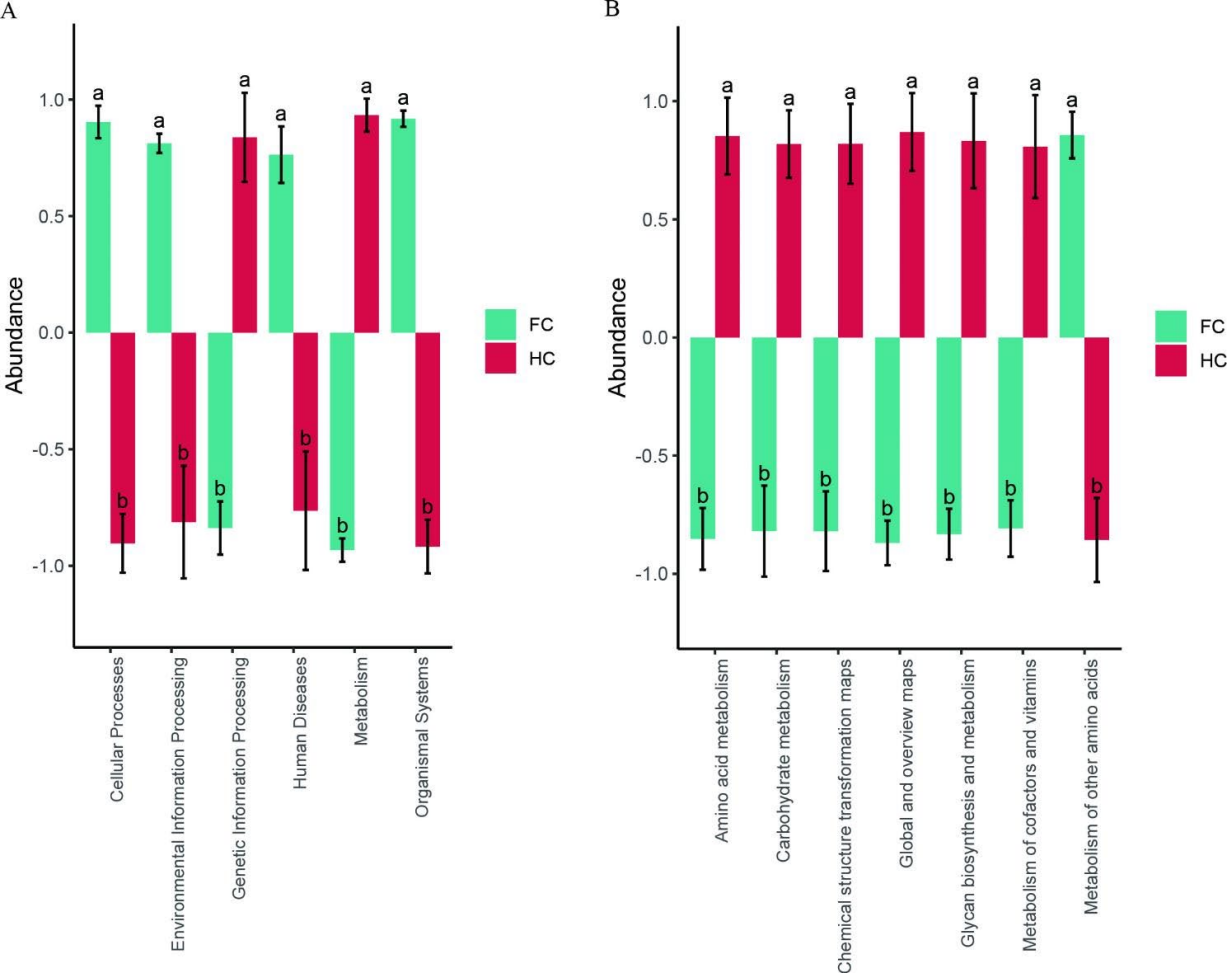
Biological metabolic pathways of soil bacteria. **(A)** KEGG-L1 pathway; **(B)** KEGG-L2 metabolic pathway. FC and HC represent soil samples from the field cultivation mode and hanging cultivation mode, respectively. Abundance standardization: The ordinate coordinate is the standardized relative abundance (the relative abundance minus the mean, divided by the standard deviation); the negative values less than the mean are displayed below, and the positive values greater than the mean are displayed above

There were significant differences in the predicted bacterial functions between FC and HC at different levels. Six primary KEGG-L1 pathways were detected in the ‘Sanghuang’ cultivation environment, wherein the abundance of the KEGG-L1 pathways including cellular processes, environmental information processing, human diseases, and organismal systems in FC was significantly higher than that in HC. The abundance of the KEGG-L1 pathways including genetic information processing and metabolism pathways in HC was significantly higher than that in FC (Fig. 7A). Similarly, significant differences between groups could also be found in the KEGG-L2 pathways detected in the ‘Sanghuang’ cultivation environment (Fig. 7B). In HC, the abundance of metabolic pathways (e.g., amino acid metabolism, carbohydrate metabolism, glycan biosynthesis and metabolism, and the metabolism of cofactors and vitamins) was significantly higher than that in FC. These results have shown that the functional composition of bacteria of ‘Sanghuang’ mushroom may be affected by different cultivation modes. No such difference, however, existed in the functional composition of fungi from the FC and HC groups.

### Correlations between soil physicochemical properties and the soil microbiome

Correlation analysis was conducted to demonstrate the relationships between soil physicochemical properties and total soil microbial communities under two cultivation modes. The results revealed that the relative abundances of both *Parafabraea* and *Serendipita* were negatively correlated with EC (*p* < 0.01), and the relative abundance of *Mycobacterium* was negatively correlated with EC (*p* < 0.001; Supplementary Fig. S2). The relative abundances of *Acinetobacter*, *Rothia*, and *Enhydrobacter* were instead positively correlated with pH (*p* < 0.01; Supplementary Fig. S2).

### Taxonomic composition of the soil microbiome

**Fig. 8:**
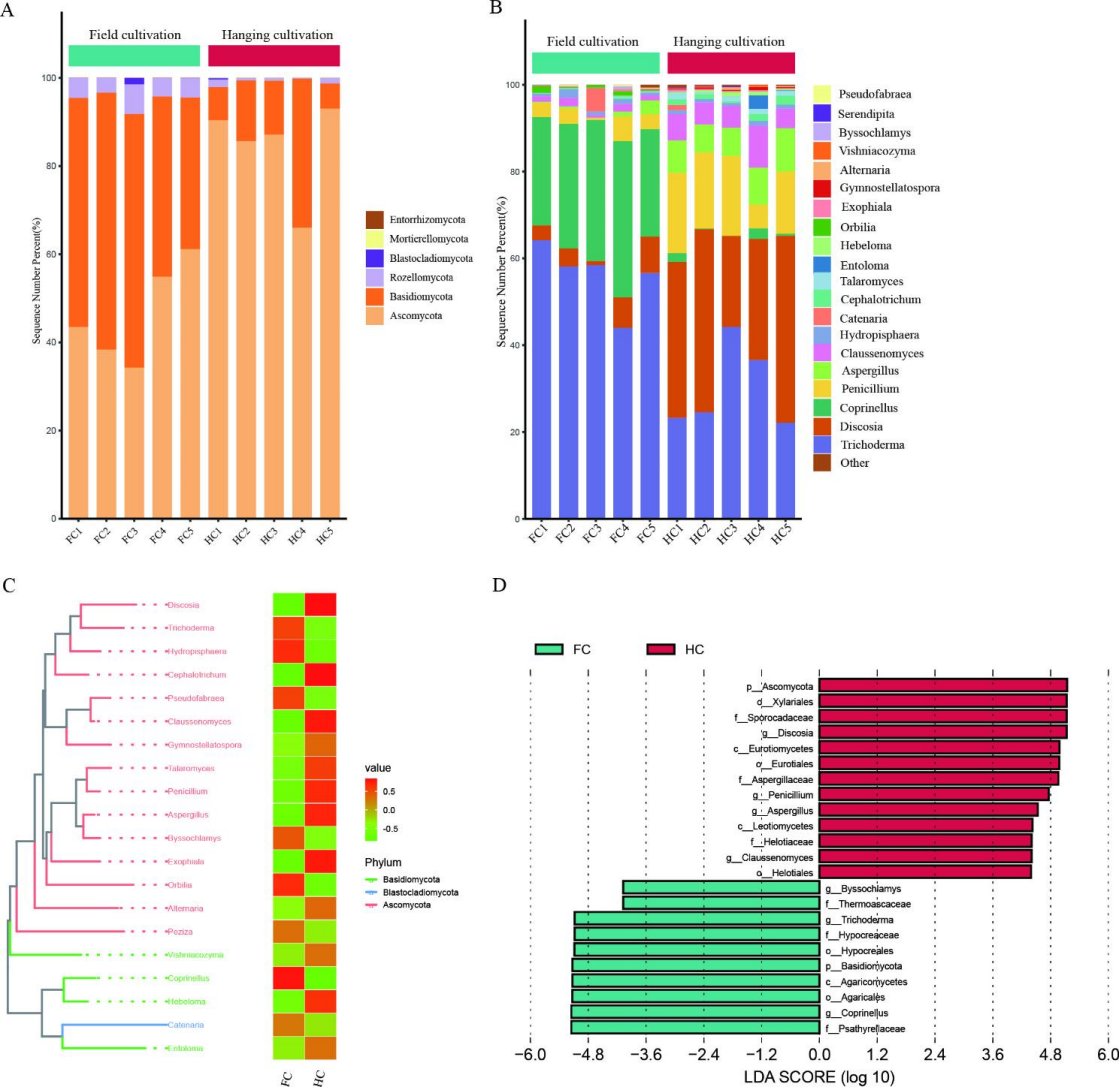
Composition differences of soil fungal communities of ‘Sanghuang’ mushroom. **(A)** fungal composition at the phylum level; **(B)** fungal composition at the genus level; **(C)** fungal phylogenetic tree; **(D)** diagram of the LDA scores calculated at the genus level between the FC and HC groups. FC and HC represent soil samples from the field cultivation mode and hanging cultivation mode, respectively; numbers 1 to 5 refer to the replicates of each sample

Based on ITS amplicon sequencing, the fungal composition and distribution were not homogeneous in the FC and HC samples. The most predominant fungal phyla were *Basidiomycota* (48.6%) and *Ascomycota* (46.5%) in the FC samples, collectively accounting for at least 95% of the total fungal community. Only the phylum *Ascomycota* was predominant in the HC samples, with a percentage greater than 84.5% (Fig. 8A). At the genus level, FC samples harbored a large proportion of *Trichoderma* (56.3%) and *Coprinellus* (29.4%), as well as smaller proportions of *Discosia* (4.8%), while HC soils harbored predominantly *Discosia* (34.0%), *Trichoderma* (30.2%), and *Penicillium* (14.9%) along with *Aspergillus* (7.8%) (Fig. 8B). Moreover, in the comparison between the FC and HC groups, *Trichoderma*, *Coprinellus*, etc., were abundant in the FC group, and *Discosia*, *Penicillium*, *Aspergillus*, etc., were abundant in the HC group (Fig. 8C-D). Differences in soil fungal composition between the two cultivation modes of ‘Sanghuang’ mushrooms were thus clearly evident. However, no such difference was observed in bacterial community composition in soil samples from different cultivation modes (Supplementary Fig. S3).

### Isolation and identity of culturable soil microorganisms

**Fig. 9:**
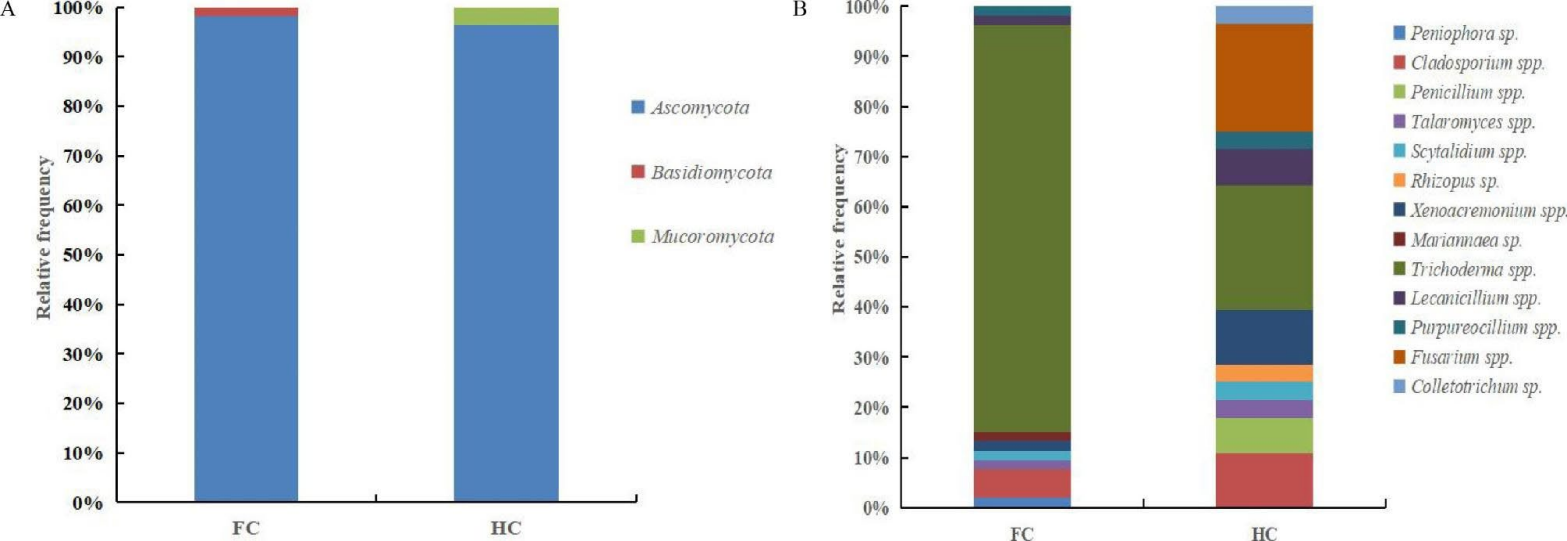
Relative abundance of culturable fungi grown under different cultivation modes at the phylum **(A)** and genus **(B)** levels. FC and HC represent soil samples from the field cultivation mode and hanging cultivation mode, respectively

Across all samples, a total of 168 isolates, including 81 fungi and 87 bacteria, were obtained from the soil samples of ‘Sanghuang’ mushroom (Supplementary Fig. S4). All culturable fungi were divided into 13 genera, which were distributed among 3 phyla, 6 classes, 6 orders, and 10 families (Supplementary Tables S5-S6), while the 87 bacterial isolates comprised 24 different genera, distributed among 4 phyla, 5 classes, 9 orders, and 15 families (Supplementary Tables S7-S8). Additionally, obvious differences in soil microbial communities, especially fungal communities, were observed between the two cultivation modes of ‘Sanghuang’ mushrooms. At the phylum level, isolates identified as *Ascomycota* and *Basidiomycota* were detected in FC soil samples, while *Ascomycota* and *Mucoromycota* were detected in HC soil samples (Fig. 9A). At the genus level, isolates belonging to the genera *Trichoderma* and *Cladosporium* were present in all soil samples, but a higher relative abundance of *Trichoderma* spp. was detected in the FC soil samples (81.13% for FC, 25.00% for HC), whereas a higher relative abundance of *Cladosporium* spp. was detected in the HC soil samples (5.66% for FC, 10.71% for HC) (Fig. 9B and Supplementary Table S6).

### Comparing culture-dependent and culture-independent approaches

A total of 168 different culturable isolates were obtained in this study. The overall number of OTUs (12,525 total) obtained from amplicon sequencing and all 168 isolates from the culture-dependent method were compared. The culture-dependent analysis showed that *Trichoderma* (61.73%) and *Bacillus* (27.59%) were the most abundant fungal and bacterial genera, respectively (Supplementary Fig. S5). In addition, members of *Cladosporium*, *Penicillium*, *Fusarium*, and *Pseudomonas* were also easily isolated. Based on the culture-independent data, sequences classified as *Trichoderma* (43.61%) and *Penicillium* (9.45%) were the major fungal genera, and *Pseudomonas* sequences made up a greater portion (15.71%) of the bacterial communities. The findings indicated that two fungal genera, *Trichoderma* and *Penicillium*, as well as one bacterial genus, *Pseudomonas*, could be obtained by both culture-dependent and culture-independent methods; thus, these genera might belong to the core soil microbes of ‘Sanghuang’ mushroom. Considering that *Trichoderma* is a common fungal pathogen of various mushrooms [10, 48] and *Trichoderma* sp. FZ0005 obtained from culture collection in this study was successfully validated by using Koch’s postulates (Supplementary Fig. S1), we concluded that *Trichoderma* was the major causal agent of ‘Sanghuang’ fungal diseases, and *Trichoderma* sp. FZ0005 was therefore used as the target for the screening of biocontrol bacteria.

### Screening of potential biocontrol strains

Among the 87 bacterial isolates, 15 antagonistic strains against *Trichoderma* sp. FZ0005 were screened, as revealed by the presence of fungal inhibition zones (Table 2 and Supplementary Fig. S6): 15 isolates (17.24% of 87) exhibited varying extents of antimicrobial activity against *Trichoderma* sp. FZ0005, with Di values ranging from 6.2 to 14.3 mm. The isolate HX0037 showed the highest antifungal activity (Di: 14.3 mm), followed by HX0016 (Di: 12.0 mm) and HX0039 (Di: 9.3 mm) (Table 2). The antagonistic strains obtained in this study could help to prevent and control ‘Sanghuang’ mushroom fungal diseases.

**Table 2:** Antifungal activity of antagonistic bacteria against the pathogen *Trichoderma* sp. FZ0005.

No.	Code	Organism	Accession number	Di (mm)
1	FX0006	*Paraburkholderia*	OP268501	7.1 ± 0.1
2	FX0012	*Bacillus*	OP268507	8.5 ± 0.1
3	FX0025	*Pseudomonas*	OP268520	8.0 ± 0.2
4	FX0033	*Stenotrophomonas*	OP268528	8.0 ± 0.1
5	HX0003	*Kosakonia*	OP268541	8.8 ± 0.6
6	HX0004	*Paraburkholderia*	OP268542	7.7 ± 0.2
7	HX0007	*Lysinibacillus*	OP268545	6.9 ± 0.1
8	HX0008	*Bacillus*	OP268546	8.1 ± 0.2
9	HX0013	*Epilithonimonas*	OP268551	8.7 ± 0.1
10	HX0016	*Bacillus*	OP268554	12.0 ± 0.1
11	HX0032	*Bacillus*	OP268570	8.6 ± 0.3
12	HX0037	*Bacillus*	OP268575	14.3 ± 0.4
13	HX0038	*Ectobacillus*	OP268576	8.7 ± 0.1
14	HX0039	*Bacillus*	OP268577	9.3 ± 0.1
15	HX0040	*Bacillus*	OP268578	6.2 ± 0.2

Note: Di, diameter of the fungal inhibition zones. Data represent the mean ± standard deviation (SD) of three replicates

## Discussion

‘Sanghuang’ mushrooms can be infected by multiple organisms, including pathogenic microbes and pests, of which mold disease caused by fungi poses the most serious challenge in industrial mushroom production. Lin et al. [9] proposed that the mold had the characteristics of a wide distribution and conidium production, which were responsible for the ‘Sanghuang’ fungal diseases. Li et al. [10] concluded that mold often appears during the cultivation of ‘Sanghuang’, which contaminates ‘Sanghuang’ and then affects its cultivation, and the specific mold types include *Trichoderma*, *Aspergillus*, and *Penicillium*. As one taxon causing fungal diseases of ‘Sanghuang’, *Trichoderma* is a great obstacle in the growth and development of ‘Sanghuang’. The biodiversity of *Trichoderma* species, such as *Trichoderma harzianum* and *Trichoderma longibrachiatum*, is very complex, with *Trichoderma harzianum* being the most widely distributed [49]. Meanwhile, the fruiting bodies of ‘Sanghuang’ mushrooms infected by *Trichoderma* showed a dark green color indicative of aggressive fungal sporulation on the surface. Additionally, part of the fruiting bodies was covered by droplets, and brown necrotic lesions even appeared. In addition to ‘Sanghuang’, *Trichoderma* diseases also appear during the cultivation of other large fruiting bodies. An et al. [50] found that *Ganoderma sichuanense*, an oriental fungus widely cultivated in northeastern China, could always be infected by *Trichoderma*. Kosanovic et al. [48] noted that crop losses could be caused by the green mold disease of *Agaricus bisporus*, and *Trichoderma* was the reason for this severe disease. There are complex interactions between soil microecosystems and crop cultivation, which may have an impact on crop disease occurrence and biomass accumulation [51]. Exploring the diversity of soil microorganisms in crop cultivation environments not only sheds light on the ecological functions they perform in the process of crop growth but also lays a foundation for the development of biocontrol resources to prevent crop diseases. The variable disease incidence of ‘Sanghuang’ mushrooms in this work is of considerable practical and scientific interest. In the present study, the soil microbiome associated with ‘Sanghuang’ mushroom suffering from fungal diseases grown under two cultivation modes (FC and HC) was characterized by the culture-dependent method and culture-independent method based on Illumina NovaSeq sequencing.

A study on the soil fungal communities of ‘Sanghuang’ mushroom performed at the genus level indicated that the most predominant genus was *Trichoderma*, regardless of the detection method (culture-dependent or culture-independent). Furthermore, the surface of ‘Sanghuang’ mushroom in the artificial Chinese medicine cultivation base showed particularly severe symptoms of *Trichoderma* infestation. Notably, our experiments also confirmed that *Trichoderma* could indeed infect mulberry trees and inhibit the growth of ‘Sanghuang’ mushrooms (Supplementary Fig. S1). Considering all these findings, the major pathogen causing fungal diseases of the ‘Sanghuang’ mushroom was *Trichoderma*. Moreover, *Penicillium* and *Fusarium* could also be detected in soil samples in our study (Supplementary Table S6). *Penicillium* spp. were detected by both culture-dependent and culture-independent methods, and *Penicillium* was also responsible for fungal diseases of other hosts [52, 53]. Notably, *Fusarium* also showed prominent pathogenicity against the host, causing a series of diseases that reduced host growth. As previously described by Hafez et al. [54], *Fusarium* can affect soybean, resulting in *Fusarium* root rot. In addition, *Fusarium* may also be the causal agent of mango leaf spots [55]. In our study, we also explored the fungal community composition on the surface of the fruiting body of ‘Sanghuang’ mushroom. Similar to the results for soil samples, a large number of molds (e.g., *Trichoderma* and *Penicillium*) were detected by high-throughput sequencing (data not shown). This suggests that other molds, including *Penicillium*, may be the agents of ‘Sanghuang’ fungal diseases.

The soil microbial community of ‘Sanghuang’ mushroom suffering from fungal diseases was shown to differ between the two cultivation modes (FC and HC). The present study showed no significant difference in soil physicochemical properties between the different cultivation modes investigated (Table 1). However, significant correlations between soil physicochemical properties and some microbes were found (Supplementary Fig. S2). This suggests that soil microorganisms were susceptible to environmental factors, which was in agreement with observations of casing soil used for cultivation of button mushroom, *Agaricus bisporus* (Lange) Imbach reported by Choudhary [56]. In addition, to reveal the biotic or abiotic factors underlying the decrease in mushroom production, the relationships between ‘Sanghuang’ mushroom physiological properties, soil physicochemical properties and soil microbial diversity must be further studied. Notably, the following significant differences in microbial community structure were observed between the FC and HC cultivation modes: the number of *Trichoderma* OTUs exhibited differences (FC > HC); the richness and diversity of microorganisms from FC were significantly lower than those from HC (for fungi, Chao: FC (439.60) < HC (594.80), Shannon: FC (5.45) < HC (6.29); for bacteria, Chao: FC (1782.47) < HC (2004.62), Shannon: FC (8.67) < HC (9.75)); the microbial correlation network exhibited differences (complexity: FC < HC); and the predicted functions of bacteria exhibited significant differences (the abundance of genetic information processing and metabolic pathways for bacteria: FC < HC). These findings reveal that cultivation mode has a strong impact on the structure of the soil microbiome of ‘Sanghuang’ mushroom suffering from fungal diseases.

Microbial communities are widely present in different environments (e.g., soil, animal intestine, and aquatic environments) [57–59]. For hosts, microbial communities are an important line of defense against pathogenic microorganism invasion from the environment [60, 61]. Numerous studies have demonstrated that the health status of a host (e.g., plant or mushroom) is a result of complex interactions between the host, the soil environment, and microorganisms, including pathogens and other microorganisms in the soil or within the plant, indicating that the richer and more diverse the microbial community in the habitats of the host is, the more complex the interspecific interactions, and the stronger the ability of the host to resist invasion by external pathogenic microorganisms [62–64]. Our findings indicated that HC samples had more OTUs, higher α-diversity, and greater microbial community complexity than FC samples. Analogous to findings for other hosts [13, 65], we speculated that there were connections between the microbial community structure and growth status of ‘Sanghuang’ mushroom.

With the emergence of limitations in traditional control methods for host diseases (e.g., chemical control and resistance breeding), the current methods of biological control for host diseases involving a search for natural antagonistic microorganisms have attracted widespread attention [43]. In recent years, the application of *Bacillus* and *Pseudomonas* as biocontrol strains has been studied in depth [66, 67]. Chen et al. [68] reported that *Bacillus subtilis* 151B1 and YBC could be used as potential biological agents to control passion fruit disease caused by *Fusarium solani*. Ren et al. [69] revealed that *Pseudomonas poae* JSU-Y1 had the potential to control the growth of toxigenic fungi in agricultural products. Our collection of bacteria antagonistic to *Trichoderma* also included members of *Bacillus* and *Pseudomonas*. Furthermore, we also performed a screening experiment of antagonistic bacteria against *Fusarium*, and the study showed that the antagonistic bacteria also included *Bacillus* with a good antifungal effect (data not shown). Notably, *Bacillus* spp. (HX0037, HX0016, and HX0039) exhibited outstanding inhibitory activity against *Trichoderma* FZ0005, suggesting that they might serve as potential biological resources for the biocontrol of ‘Sanghuang’ diseases. As reported in other studies, the use of biofungicides based on *Bacillus* species could be regarded as a biological alternative to synthetic fungicides employed in mushroom production [20, 70]. We suggest that a bacterial suspension of *Bacillus* strains with antifungal activity against *Trichoderma* FZ0005 should be sprayed onto the cultivation soil or the surface of bag-cultivated ‘Sanghuang’ mushrooms during propagation and at the stage of fruiting body formation. Moreover, adding beneficial *Bacillus* (HX0037, HX0016, and HX0039) bacteria to water tanks used for daily irrigation might provide an additional strategy for disease management. The biocontrol activity and mechanism of these antagonistic bacteria, however, remain to be further characterized. According to our findings, we hypothesized that *Bacillus* occupied a dominant ecological niche in the soil microbiome of HC, which not only stabilized the soil microecosystem but also effectively antagonized the major pathogen causing the fungal diseases of ‘Sanghuang’, thereby possibly reducing the production of mold conidia released into the air.

## Conclusions

High-throughput sequencing technology and traditional culture-dependent methods were used to analyze the structure of soil microbial communities of ‘Sanghuang’ mushroom suffering from fungal diseases. Our results revealed that cultivation mode could influence the structure of soil microbial communities of ‘Sanghuang’ mushroom, and the *Trichoderma* genus was the major causal agent of ‘Sanghuang’ fungal diseases. Three *Bacillus* spp. (HX0037, HX0016, and HX0039) exhibited effective antifungal activity against *Trichoderma* sp. FZ0005 and might be useful as future biocontrol agents against fungal diseases affecting ‘Sanghuang’ mushroom.

### Electronic supplementary material

Below is the link to the electronic supplementary material.

Supplementary Material 1: **Table S1** High-throughput sequencing statistics of soil samples of ‘Sanghuang’ mushroom. **Table S2.** α-Diversity index of soil samples from the high-throughput sequencing data. **Table S3.** PERMANOVA results from the high-throughput sequencing data. **Table S4.** Correlation network analysis of soil microbial communities from the high-throughput sequencing data. **Table S5.** Distribution of culturable fungi isolated from the soil of ‘Sanghuang’ mushroom. **Table S6.** Relative frequency of culturable fungi in the soil samples grown under different cultivation modes of ‘Sanghuang’ mushroom. **Table S7.** Distribution of culturable bacteria isolated from the soil associated with ‘Sanghuang’ mushroom. **Table S8.** Relative frequency of culturable bacteria in the soil samples grown under different cultivation modes of ‘Sanghuang’ mushroom. Fig. S1 Pathogenicity determination of the pathogenic fungus *Trichoderma* FZ0005. **Fig. S2** Correlations between soil physicochemical properties and total soil microbial taxa associated with ‘Sanghuang’ mushroom. **Fig. S3** Composition of soil bacterial communities of ‘Sanghuang’ mushroom. **Fig. S4** Colony features of some culturable soil microbes and electrophoretograms of their PCR products. **Fig. S5** Comparison of genus-level microbial composition between culture-dependent and culture-independent methods. **Fig. S6** Screening of partial antagonistic strains against *Trichoderma* sp. FZ0005.

## Data Availability

All data are available upon request to the corresponding author. Complete culture-independent sequence datasets were submitted to the NCBI Sequence Read Archive (SRA) database under the accession number SRP395845 (ITS) and SRP395839 (16 S rDNA), respectively. The data can be found at https://www.ncbi.nlm.nih.gov/sra/?term=SRP395845 (ITS) and https://www.ncbi.nlm.nih.gov/sra/?term=SRP395839 (16 S rDNA). The ITS gene sequences of the culturable fungal isolates were submitted to GenBank under the accession numbers OP269747-OP269827. The 16 S rDNA sequences of the culturable bacterial isolates were submitted to GenBank under the accession numbers OP268496-OP268582. All data generated or analyzed during this study are included in this article and its supporting information files.

## Abbreviations

FC: Field cultivation
HC: Hanging cultivation
OTUs: Operational taxonomic units
EC: Electrical conductivity
AN: Available nitrogen
AP: Available phosphorus
AK: Available potassium
PCR: Polymerase chain reaction
WA: Water agar
GA: Gause’s agar
PDA: Potato dextrose agar
PCoA: Principal coordinate analysis
PERMANOVA: Permutational multivariate analysis of variance
Di: Diameter of the fungal inhibition zone
