# Supplementary material for: Structure and ecological function of the soil microbiome associated with ‘Sanghuang’ mushrooms suffering from fungal diseases
Source: BMC Microbiol. 2023 Aug 12;23:218. doi: 10.1186/s12866-023-02965-z (PMC10422728; doi:10.1186/s12866-023-02965-z)
Supplement: Supplementary file 1 — Supplementary Material 1: Table S1 High-throughput sequencing statistics of soil samples of ‘Sanghuang’ mushroom. Table S2. α-Diversity index of soil samples from the high-throughput sequencing data. Table S3. PERMANOVA results from the high-throughput sequencing data. Table S4. Correlation network analysis of soil microbial communities from the high-throughput sequencing data. Table S5. Distribution of culturable fungi isolated from the soil of ‘Sanghuang’ mushroom. Table S6. Relative frequency of culturable fungi in the soil samples grown under different cultivation modes of ‘Sanghuang’ mushroom. Table S7. Distribution of culturable bacteria isolated from the soil associated with ‘Sanghuang’ mushroom. Table S8. Relative frequency of culturable bacteria in the soil samples grown under different cultivation modes of ‘Sanghuang’ mushroom. Fig. S1 Pathogenicity determination of the pathogenic fungus Trichoderma FZ0005. Fig. S2 Correlations between soil physicochemical properties and total soil microbial taxa associated with ‘Sanghuang’ mushroom. Fig. S3 Composition of soil bacterial communities of ‘Sanghuang’ mushroom. Fig. S4 Colony features of some culturable soil microbes and electrophoretograms of their PCR products. Fig. S5 Comparison of genus-level microbial composition between culture-dependent and culture-independent methods. Fig. S6 Screening of partial antagonistic strains against Trichoderma sp. FZ0005. [file 12866_2023_2965_MOESM1_ESM.docx]

**Table S1**

High-throughput sequencing statistics of soil samples of ‘Sanghuang’ mushroom.

|  | fungi | | bacteria | |
| --- | --- | --- | --- | --- |
| sample | original sequences | effective sequences | original sequences | effective sequences |
| HC1 | 92225 | 75483 | 78884 | 43339 |
| HC2 | 87059 | 74022 | 88672 | 56736 |
| HC3 | 85419 | 72135 | 88184 | 55839 |
| HC4 | 82867 | 70468 | 88654 | 54270 |
| HC5 | 83986 | 66492 | 86863 | 56118 |
| FC1 | 79691 | 67527 | 81659 | 55909 |
| FC2 | 77351 | 66375 | 85526 | 56256 |
| FC3 | 84571 | 73661 | 87599 | 52781 |
| FC4 | 84982 | 74063 | 77383 | 48308 |
| FC5 | 84356 | 70964 | 88546 | 54910 |

Note: FC and HC represent soil samples from field cultivation mode and hanging cultivation mode, respectively; numbers 1 to 5 refer to the replicates of each sample.

**Table S2**

α-Diversity index of soil samples from the high-throughput sequencing data.

| Group | Chao | Shannon |
| --- | --- | --- |
| fungi |  |  |
| FC | 439.60±83.73 | 5.45±0.31 |
| HC | 594.80±55.34 | 6.29±0.24 |
| *p* | 0.009 | 0.001 |
| bacteria |  |  |
| FC | 1782.47±122.45 | 8.67±0.20 |
| HC | 2004.62±130.93 | 9.75±0.11 |
| *p* | 0.024 | 0.000 |

Note: Data represent the mean ± standard deviation (SD) of two independent replicates. Means followed by different letters are significantly different at *p* < 0.05.

**Table S3**

PERMANOVA results from the high-throughput sequencing data.

|  | Soil Type | pseudo-*F* | *p*-value |
| --- | --- | --- | --- |
| fungi | FC & HC | 9.294 | 0.007 |
| bacteria | FC & HC | 15.563 | 0.008 |

Note: FC and HC represent soil samples from field cultivation mode and hanging cultivation mode, respectively.

**Table S4**

Correlation network analysis of soil microbial communities from the high-throughput sequencing data.

|  | fungi | | bacteria | |
| --- | --- | --- | --- | --- |
|  | FC | HC | FC | HC |
| Number of nodes | 30 | 30 | 30 | 30 |
| Number of edges | 23 | 25 | 44 | 49 |
| Positive edges | 14 | 20 | 26 | 25 |
| Negative edges | 9 | 5 | 18 | 24 |

Note: FC and HC represent soil samples from field cultivation mode and hanging cultivation mode, respectively.

**Table S5**

Distribution of culturable fungi isolated from the soil of ‘Sanghuang’ mushroom.

| Phyla | Classes | Orders | Families | Genera |
| --- | --- | --- | --- | --- |
|  |  |  |  |  |
| Basidiomycota (1) | Agaricomycetes (1) | Russulales (1) | Peniophoraceae (1) | *Peniophora* sp. (1) |
| Ascomycota (79) | Dothideomycetes (6) | Cladosporiales (6) | Cladosporiaceae (6) | *Cladosporium* spp. (6) |
|  | Eurotiomycetes (4) | Eurotiales (4) | Aspergillaceae (2) | *Penicillium* spp. (2) |
|  |  |  | Trichocomaceae (2) | *Talaromyces* spp. (2) |
|  | Leotiomycetes (2) | / | / | *Scytalidium* spp. (2) |
|  | Sordariomycetes (67) | Glomerellales (1) | Glomerellaceae (1) | *Colletotrichum* sp. (1) |
|  |  | Hypocreales (66) | Cordycipitaceae (3) | *Lecanicillium* spp. (3) |
|  |  |  | Hypocreaceae (50) | *Trichoderma* spp. (50) |
|  |  |  | Nectriaceae (11) | *Fusarium* spp. (6) |
|  |  |  |  | *Mariannaea* sp. (1) |
|  |  |  |  | *Xenoacremonium* spp. (4) |
|  |  |  | Ophiocordycipitaceae (2) | *Purpureocillium* spp. (2) |
| Mucoromycota (1) | Mucoromycetes (1) | Mucorales (1) | Rhizopodaceae (1) | *Rhizopus* sp. (1) |

**Table S6**

Relative frequency of culturable fungi in the soil samples grown under different cultivation modes of ‘Sanghuang’ mushroom.

| Taxons | | Relative frequency (%) | | Total |
| --- | --- | --- | --- | --- |
|  |  | FC | HC |  |
| Ⅰ | Agaricomycetes |  |  |  |
| 1 | *Peniophora* sp. | 1.89% | 0.00% | 1.23% |
| Ⅱ | Dothideomycetes |  |  |  |
| 2 | *Cladosporium* spp. | 5.66% | 10.71% | 7.41% |
| Ⅲ | Eurotiomycetes |  |  |  |
| 3 | *Penicillium* spp. | 0.00% | 7.14% | 2.47% |
| 4 | *Talaromyces* spp. | 1.89% | 3.57% | 2.47% |
| Ⅳ | Leotiomycetes |  |  |  |
| 5 | *Scytalidium* spp. | 1.89% | 3.57% | 2.47% |
| Ⅴ | Mucoromycetes |  |  |  |
| 6 | *Rhizopus* sp*.* | 0.00% | 3.57% | 1.23% |
| Ⅵ | Sordariomycetes |  |  |  |
| 7 | *Xenoacremonium* spp. | 1.89% | 10.71% | 4.94% |
| 8 | *Mariannaea* sp. | 1.89% | 0.00% | 1.23% |
| 9 | *Trichoderma* spp. | 81.13% | 25.00% | 61.73% |
| 10 | *Lecanicillium* spp. | 1.89% | 7.14% | 3.70% |
| 11 | *Purpureocillium* spp. | 1.89% | 3.57% | 2.47% |
| 12 | *Fusarium* spp. | 0.00% | 21.43% | 7.41% |
| 13 | *Colletotrichum* sp. | 0.00% | 3.57% | 1.23% |
| Total | | 100.00% | 100.00% | 100.00% |

Note: FC and HC represent soil samples from field cultivation mode and hanging cultivation mode, respectively.

**Table S7**

Distribution of culturable bacteria isolated from the soil associated with ‘Sanghuang’ mushroom.

| Phyla | Classes | Orders | Families | Genera |
| --- | --- | --- | --- | --- |
|  |  |  |  |  |
| Actinobacteria (7) | Actinobacteria (7) | Corynebacteriales (1) | Nocardiaceae (1) | *Rhodococcus* sp. (1) |
|  |  | Micrococcales (6) | Brevibacteriaceae (4) | *Brevibacterium* spp. (4) |
|  |  |  | Microbacteriaceae (2) | *Microbacterium* spp. (2) |
| Firmicutes (38) | Bacilli (38) | Bacillales (38) | Bacillaceae (34) | *Bacillus* spp. (24) |
|  |  |  |  | *Ectobacillus* sp. (1) |
|  |  |  |  | *Lysinibacillus* spp. (3) |
|  |  |  |  | *Priestia* spp. (6) |
|  |  |  | Paenibacillaceae (1) | *Paenibacillus* sp. (1) |
|  |  |  | Staphylococcaceae (3) | *Mammaliicoccus* spp. (2) |
|  |  |  |  | *Staphylococcus* sp. (1) |
| Proteobacteria (37) | Betaproteobacteria (5) | Burkholderiales (5) | Burkholderiaceae (5) | *Burkholderia* spp. (2) |
|  |  |  |  | *Paraburkholderia* spp. (3) |
|  | Gammaproteobacteria (32) | Enterobacterales (16) | Enterobacteriaceae (14) | *Citrobacter* sp. (1) |
|  |  |  |  | *Klebsiella* spp. (2) |
|  |  |  |  | *Kosakonia* spp. (4) |
|  |  |  |  | *Raoultella* spp. (7) |
|  |  |  | Erwiniaceae (1) | *Izhakiella* sp. (1) |
|  |  |  | Morganellaceae (1) | *Providencia* sp. (1) |
|  |  | Moraxellales (2) | Moraxellaceae (2) | *Acinetobacter* spp. (2) |
|  |  | Pseudomonadales (10) | Pseudomonadaceae (10) | *Pseudomonas* spp. (10) |
|  |  | Xanthomonadales (4) | Rhodanobacteraceae (2) | *Dyella* spp. (2) |
|  |  |  | Xanthomonadaceae (2) | *Stenotrophomonas* spp. (2) |
| Bacteroidetes (5) | Flavobacteriia (5) | Flavobacteriales (5) | Weeksellaceae (5) | *Chryseobacterium* spp. (4) |
|  |  |  |  | *Epilithonimonas* sp. (1) |

**Table S8**

Relative frequency of culturable bacteria in the soil samples grown under different cultivation modes of ‘Sanghuang’ mushroom.

| Taxons | | Relative frequency (%) | | Total |
| --- | --- | --- | --- | --- |
|  |  | FC | HC |  |
| Ⅰ | Actinobacteria |  |  |  |
| 1 | *Brevibacterium* spp. | 4.65% | 4.55% | 4.60% |
| 2 | *Microbacterium* spp. | 0.00% | 4.55% | 2.30% |
| 3 | *Rhodococcus* sp. | 2.33% | 0.00% | 1.15% |
| Ⅱ | Bacilli |  |  |  |
| 4 | *Bacillus* spp. | 25.58% | 29.55% | 27.59% |
| 5 | *Ectobacillus* sp. | 0.00% | 2.27% | 1.15% |
| 6 | *Lysinibacillus* spp. | 2.33% | 4.55% | 3.45% |
| 7 | *Mammaliicoccus* spp. | 4.65% | 0.00% | 2.30% |
| 8 | *Paenibacillus* sp. | 2.33% | 0.00% | 1.15% |
| 9 | *Priestia* spp. | 6.98% | 6.82% | 6.90% |
| 10 | *Staphylococcus* sp. | 2.33% | 0.00% | 1.15% |
| Ⅲ | Betaproteobacteria |  |  |  |
| 11 | *Burkholderia* spp. | 2.33% | 2.27% | 2.30% |
| 12 | *Paraburkholderia* spp. | 4.65% | 2.27% | 3.45% |
| Ⅳ | Flavobacteriia |  |  |  |
| 13 | *Chryseobacterium* spp. | 6.98% | 2.27% | 4.60% |
| 14 | *Epilithonimonas* sp. | 0.00% | 2.27% | 1.15% |
| Ⅴ | Gammaproteobacteria |  |  |  |
| 15 | *Acinetobacter* spp. | 0.00% | 4.55% | 2.30% |
| 16 | *Citrobacter* sp. | 0.00% | 2.27% | 1.15% |
| 17 | *Dyella* spp. | 2.33% | 2.27% | 2.30% |
| 18 | *Izhakiella* sp. | 2.33% | 0.00% | 1.15% |
| 19 | *Kosakonia* spp. | 0.00% | 9.09% | 4.60% |
| 20 | *Klebsiella* spp. | 4.65% | 0.00% | 2.30% |
| 21 | *Providencia* sp. | 0.00% | 2.27% | 1.15% |
| 22 | *Pseudomonas* spp. | 23.26% | 0.00% | 11.49% |
| 23 | *Raoultella* spp. | 0.00% | 15.91% | 8.05% |
| 24 | *Stenotrophomonas* spp. | 2.33% | 2.27% | 2.30% |
| Total | | 100.00% | 100.00% | 100.00% |

Note: FC and HC represent soil samples from field cultivation mode and hanging cultivation mode, respectively.


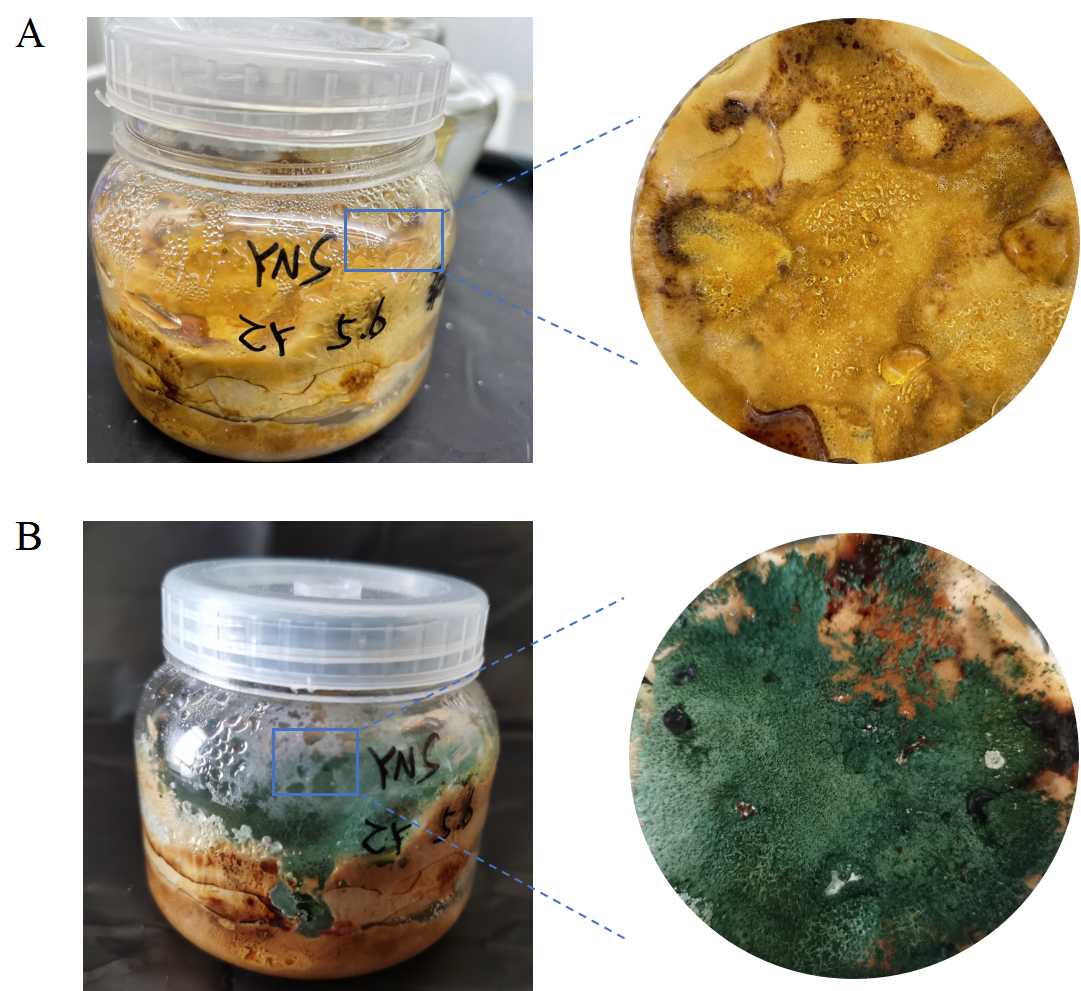


**Fig. S1** Pathogenicity determination of the pathogenic fungus *Trichoderma* FZ0005. (**A**) ‘Sanghuang’ strain without inoculation of FZ0005; (**B**) ‘Sanghuang’ strain with inoculation of FZ0005.


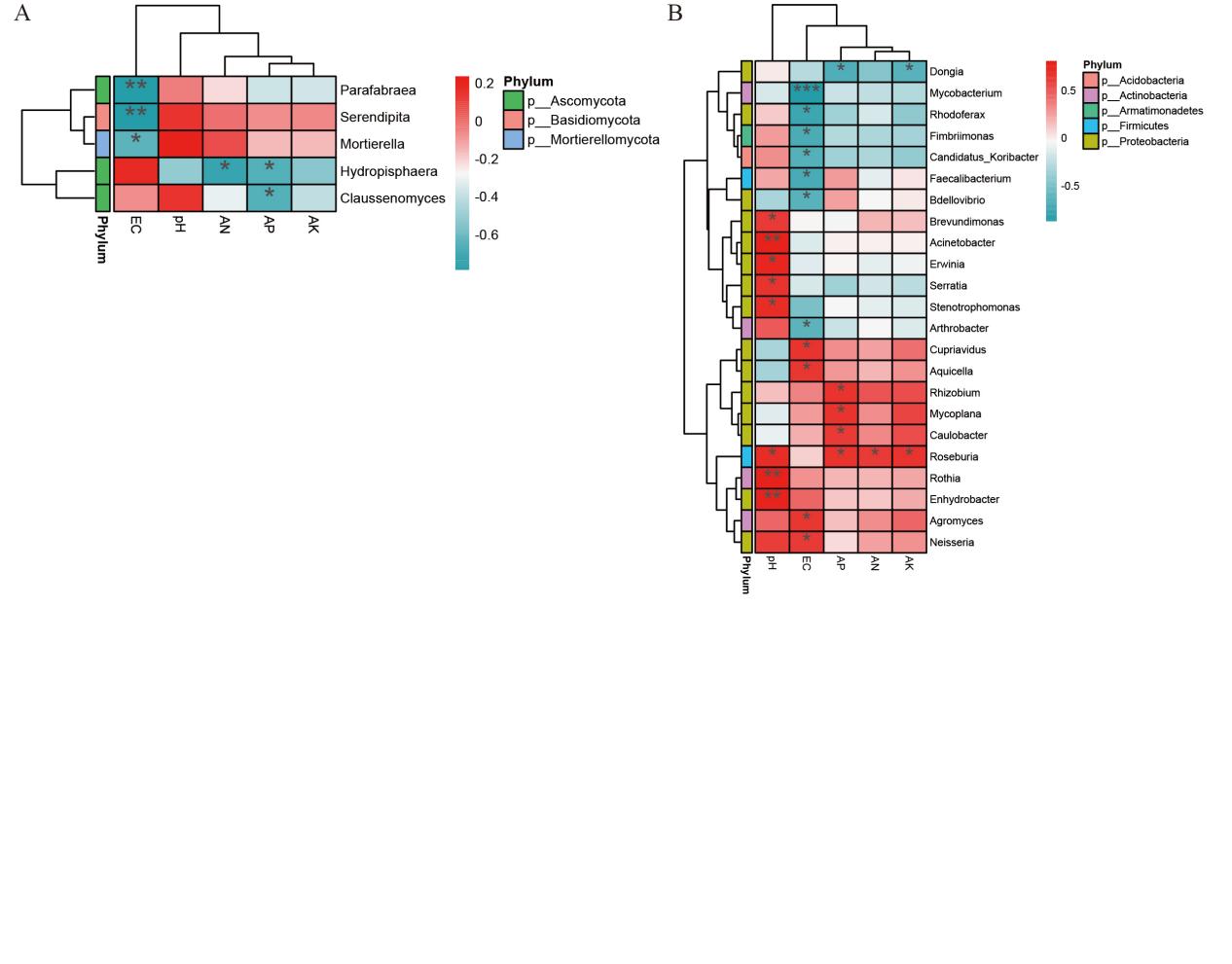


**Fig. S2** Correlations between soil physicochemical properties and total soil microbial taxa associated with ‘Sanghuang’ mushroom. (**A**) fungi; (**B**) bacteria. * represents ‘*p* < 0.05’, ** represents ‘*p* < 0.01’, *** represents ‘*p* < 0.001’. EC, pH, AN, AP and AK represent electrical conductivity, potential of hydrogen, available nitrogen, available phosphorus, and available potassium, respectively.


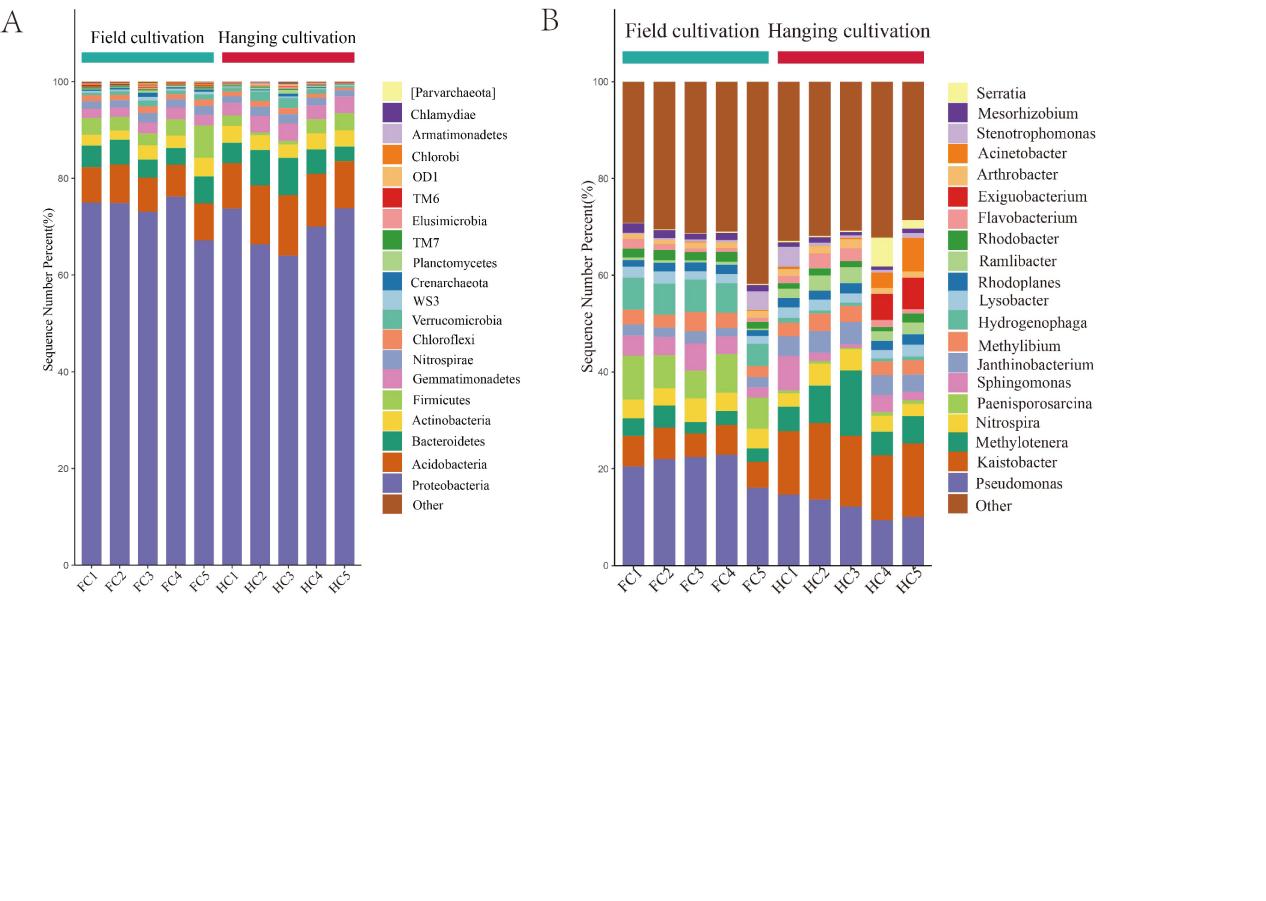


**Fig. S3** Composition of soil bacterial communities of ‘Sanghuang’ mushroom. (**A**) at the phylum level; (**B**) at the genus level. FC and HC represent soil samples from field cultivation mode and hanging cultivation mode, respectively; numbers 1 to 5 refer to the replicates of each sample.


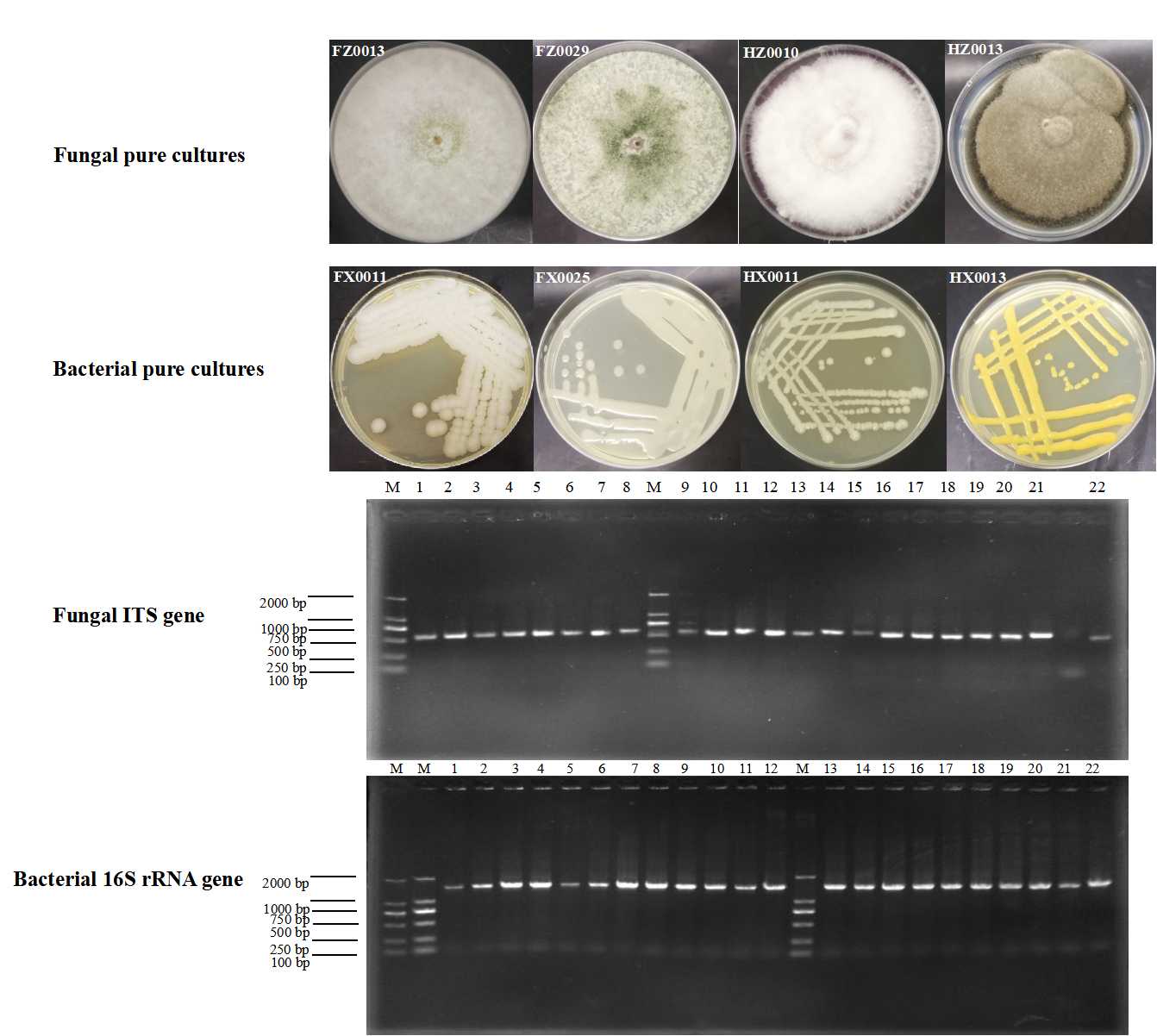


**Fig. S4** Colony features of some culturable soil microbes and electrophoretograms of their PCR products. M indicates the marker (DNA Ladder). 1-22 indicate 22 strains randomly selected from all cultivable soil fungi or bacteria.


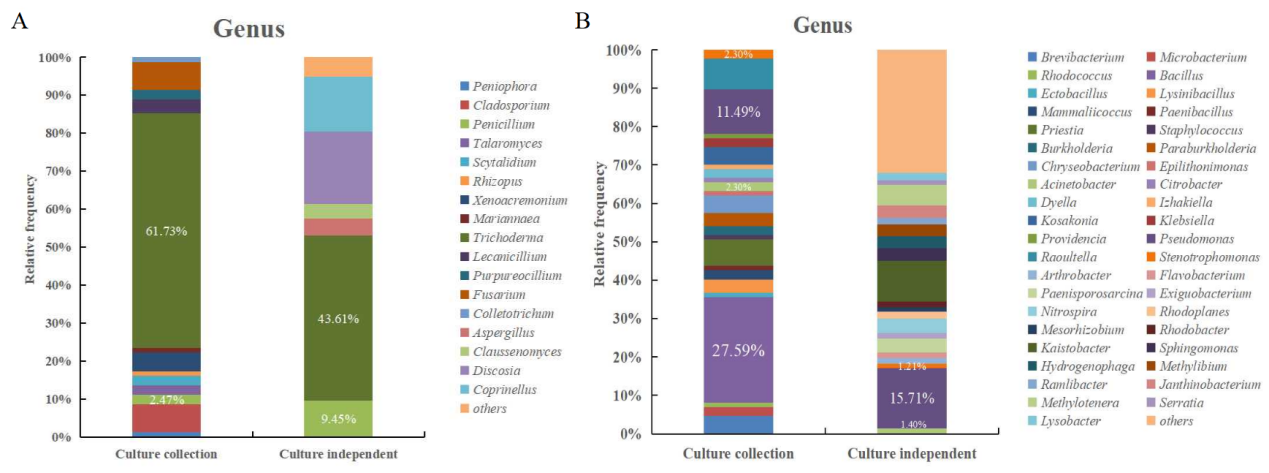


**Fig. S5** Comparison of genus-level microbial composition between culture-dependent and culture-independent methods. (**A**) fungi; (**B**) bacteria. Culture collection, taxonomic composition of the 168 strains isolated from soil of ‘Sanghuang’ mushroom; Culture-independent method, taxonomic composition of OTUs detected in soil samples used for the culture-dependent method, and the genera with a relative abundance < 1% are included in ‘others’.


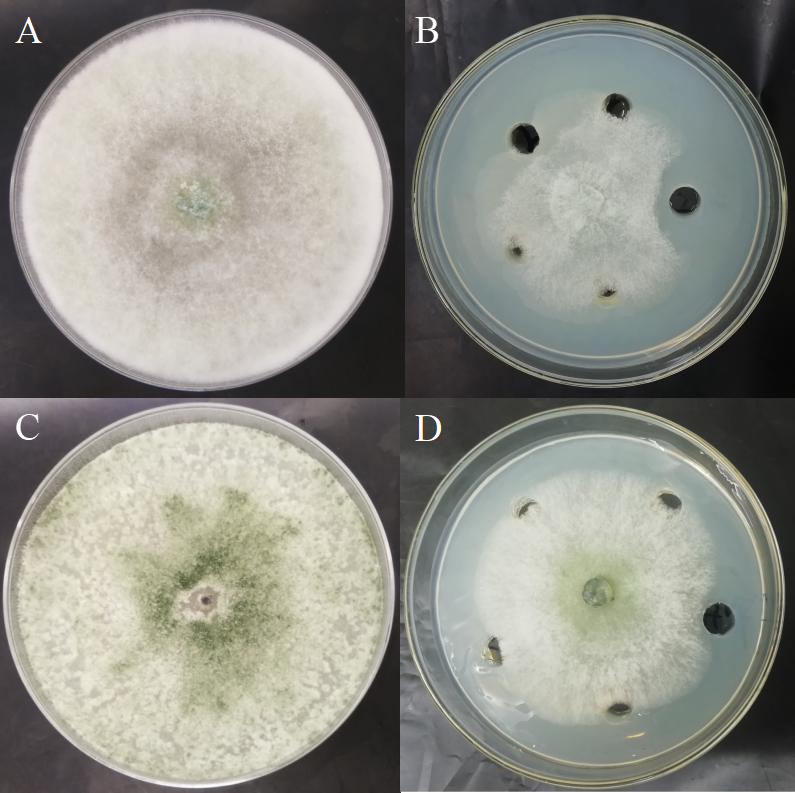


**Fig. S6** Screening of partial antagonistic strains against *Trichoderma* sp. FZ0005. (**A**)

*Trichoderma* sp. FZ0005 without inoculation of bacterial cultures; (**B**) *Trichoderma* sp. FZ0005 with inoculation of bacterial cultures.
